# Isolation, characterization, anti-MRSA evaluation, and in-silico multi-target anti-microbial validations of actinomycin X_2_ and actinomycin D produced by novel *Streptomyces smyrnaeus* UKAQ_23

**DOI:** 10.1038/s41598-021-93285-7

**Published:** 2021-07-15

**Authors:** Kamal A. Qureshi, Avinash D. Bholay, Pankaj K. Rai, Hamdoon A. Mohammed, Riaz A. Khan, Faizul Azam, Mariusz Jaremko, Abdul-Hamid Emwas, Piotr Stefanowicz, Mateusz Waliczek, Monika Kijewska, Ehab A. Ragab, Medhat Rehan, Gamal O. Elhassan, Md Jamir Anwar, Dinesh K. Prajapati

**Affiliations:** 1 Faculty of Biosciences and Biotechnology, Invertis University, Bareilly, UP 243123 India; 2 Department of Pharmaceutics, Unaizah College of Pharmacy, Qassim University, Unaizah, Qassim 51911 Saudi Arabia; 3 Department of Microbiology, KTHM College, Savitribai Phule Pune University, Nashik, MS 422002 India; 4 Department of Medicinal Chemistry and Pharmacognosy, College of Pharmacy, Qassim University, Buraydah, Qassim 51452 Saudi Arabia; 5 Department of Pharmaceutical Chemistry and Pharmacognosy, Unaizah College of Pharmacy, Qassim University, Unaizah, Qassim 51911 Saudi Arabia; 6 Biological and Environmental Sciences and Engineering Division (BESE) Division, King Abdullah University of Science and Technology (KAUST), Thuwal, 23955-6900 Saudi Arabia; 7 Core Labs, King Abdullah University of Science and Technology (KAUST), Thuwal, 23955-6900 Saudi Arabia; 8 Faculty of Chemistry, University of Wroclaw, F. Joliot-Curie, Street-14, 50-383 Wroclaw, Poland; 9 Department of Pharmacognosy, Faculty of Pharmacy, Al-Azhar University, Cairo, 11371 Egypt; 10 Department of Genetics, Faculty of Agriculture, Kafr El-Sheikh University, Kafr El-Sheikh, 33516 Egypt; 11 Department of Plant Production and Protection, College of Agriculture and Veterinary Medicine, Qassim University, Buraydah, Qassim 51452 Saudi Arabia; 12 Department of Pharmacology and Toxicology, Unaizah College of Pharmacy, Qassim University, Unaizah, Qassim 51911 Saudi Arabia

**Keywords:** Antimicrobials, Applied microbiology, Soil microbiology, Biotechnology, Drug discovery

## Abstract

*Streptomyces smyrnaeus* UKAQ_23, isolated from the mangrove-sediment, collected from Jubail,Saudi Arabia, exhibited substantial antimicrobial activity against methicillin-resistant *Staphylococcus aureus* (MRSA), including non-MRSA Gram-positive test bacteria. The novel isolate, under laboratory-scale conditions, produced the highest yield (561.3 ± 0.3 mg/kg fermented agar) of antimicrobial compounds in modified ISP-4 agar at pH 6.5, temperature 35 °C, inoculum 5% v/w, agar 1.5% w/v, and an incubation period of 7 days. The two major compounds, K_1_ and K_2_, were isolated from fermented medium and identified as Actinomycin X_2_ and Actinomycin D, respectively, based on their structural analysis. The antimicrobial screening showed that Actinomycin X_2_ had the highest antimicrobial activity compared to Actinomycin D, and the actinomycins-mixture (X_2_:D, 1:1, w/w) against MRSA and non-MRSA Gram-positive test bacteria, at 5 µg/disc concentrations. The MIC of Actinomycin X_2_ ranged from 1.56–12.5 µg/ml for non-MRSA and 3.125–12.5 µg/ml for MRSA test bacteria. An in-silico molecular docking demonstrated isoleucyl tRNA synthetase as the most-favored antimicrobial protein target for both actinomycins, X_2_ and D, while the penicillin-binding protein-1a, was the least-favorable target-protein. In conclusion, *Streptomyces smyrnaeus* UKAQ_23 emerged as a promising source of Actinomycin X_2_ with the potential to be scaled up for industrial production, which could benefit the pharmaceutical industry.

## Introduction

Nosocomial infections caused by multi-drug resistant (MDR) pathogens, including MRSA, vancomycin-resistant *Staphylococcus aureus* (VRSA), vancomycin-resistant *Enterococci* (VRE), *Pseudomonas aeruginosa*, *Acinetobacter baumannii*, *Escherichia coli*, *Klebsiella pneumonia*, and *Enterobacter* spp., are one of the major causes of death among hospitalized patients^1^. The resistance to most conventional antibiotics, *i.e.*, second-and third-generation cephalosporins, fluoroquinolones, penicillin combined with a beta-lactamase inhibitor, and carbapenems^2^, form this list. Owing to the presence of extremely resistant bacteria, curing antibiotic-resistant nosocomial infections presents an alarming situation for public healthcare. This necessitates the discovery of new antimicrobial agents to cope with the life-threatening infections caused by MDR pathogens^3,4^. Microbes strive against fellow microbes for their existence because of limited space and nutrients in the natural ecosystem. Therefore, microbes generally develop several strategies for surviving in their environment, one of which is the production of antimicrobial agents^5^. Actinomycetes, a well-known, invaluable bacterial group found in multiple habitats, is responsible for producing a wide range of industrially valuable and medically significant compounds^6–9^. *Streptomyces,* a commercially invaluable and medically significant actinomycetes genus, produces a wide range of biologically active compounds, *i.e*., antibiotics, anticancer, antiviral, herbicidal, and insecticidal agents and that has kept the focus further for continuing studies on this genus and its products^10–12^. The discovery rate of novel bioactive compounds from the soil actinomycetes has decreased over the years, while the rate of re-isolation of known bioactive compounds has increased^13^. Many studies, however, indicated that the mangrove soil is still the primary source for the isolation of novel bioactive actinomycetes^1,2,6,13–16^. To prevent re-isolation of the same species and antibiotics, the target soil sample must be very precise. A mangrove sediment area, found in Jubail, Saudi Arabia, possessed a large, unexplored region that could be an opulent source for the isolation of novel actinomycetes. Therefore, the present study aimed to isolate the novel antibiotic-producing actinomycetes from the mangrove sediment samples of Jubail, Saudi Arabia. The current research demonstrated the isolation, identification, antimicrobial screening of a novel actinomycete strain, *Streptomyces smyrnaeus* UKAQ_23, along with the production, fermentation-optimization, isolation, purification, structure elucidation, and antimicrobial screening of the antimicrobial compounds produced by strain UKAQ_23. An in-silico molecular docking of isolated antibiotics was performed against various antimicrobial target proteins. A detailed intermolecular interaction analysis with the binding energies and docking feasibility was undertaken.

## Results

### Isolation and preliminary antimicrobial screening of actinomycetes

Five actinomycetes strains, namely UKAQ_04, UKAQ_05, UKAQ_07, UKAQ_09, and UKAQ_23, were isolated from the twenty-five collected mangrove sediment samples (see, Supplementary Figure S1).

**Figure 1:**
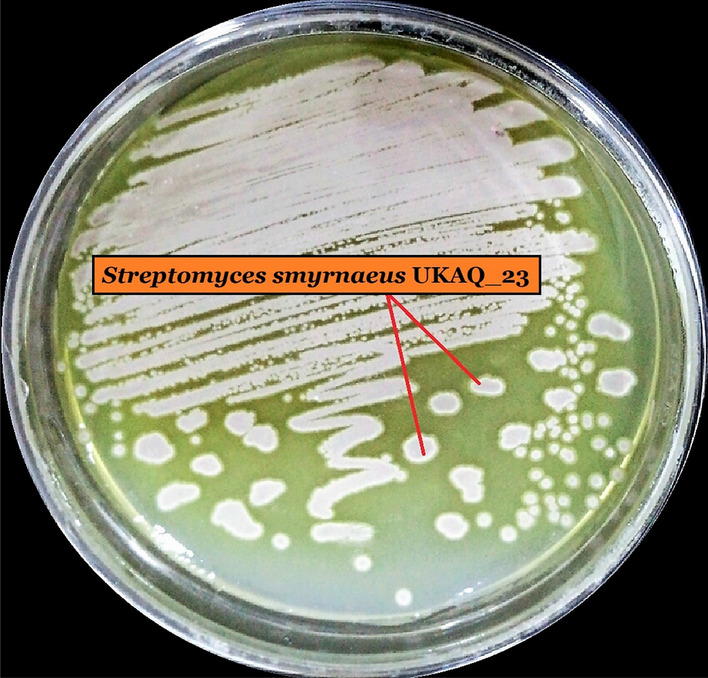
Growth of UKAQ_23 on modified International Streptomyces Project (ISP)-4 agar at 35 °C for 7 days.

Out of the five isolated actinomycetes, only one strain, UKAQ_23, exhibited substantial antimicrobial activity against 10 Gram-positive test bacteria, including 03 MRSA strains, while no antimicrobial activity was observed against 07 Gram-negative test bacteria and 02 fungal strains. Therefore, strain UKAQ_23 was selected for further studies (Fig. 1).

### Identification and characterization of strain UKAQ_23

**Figure 2:**
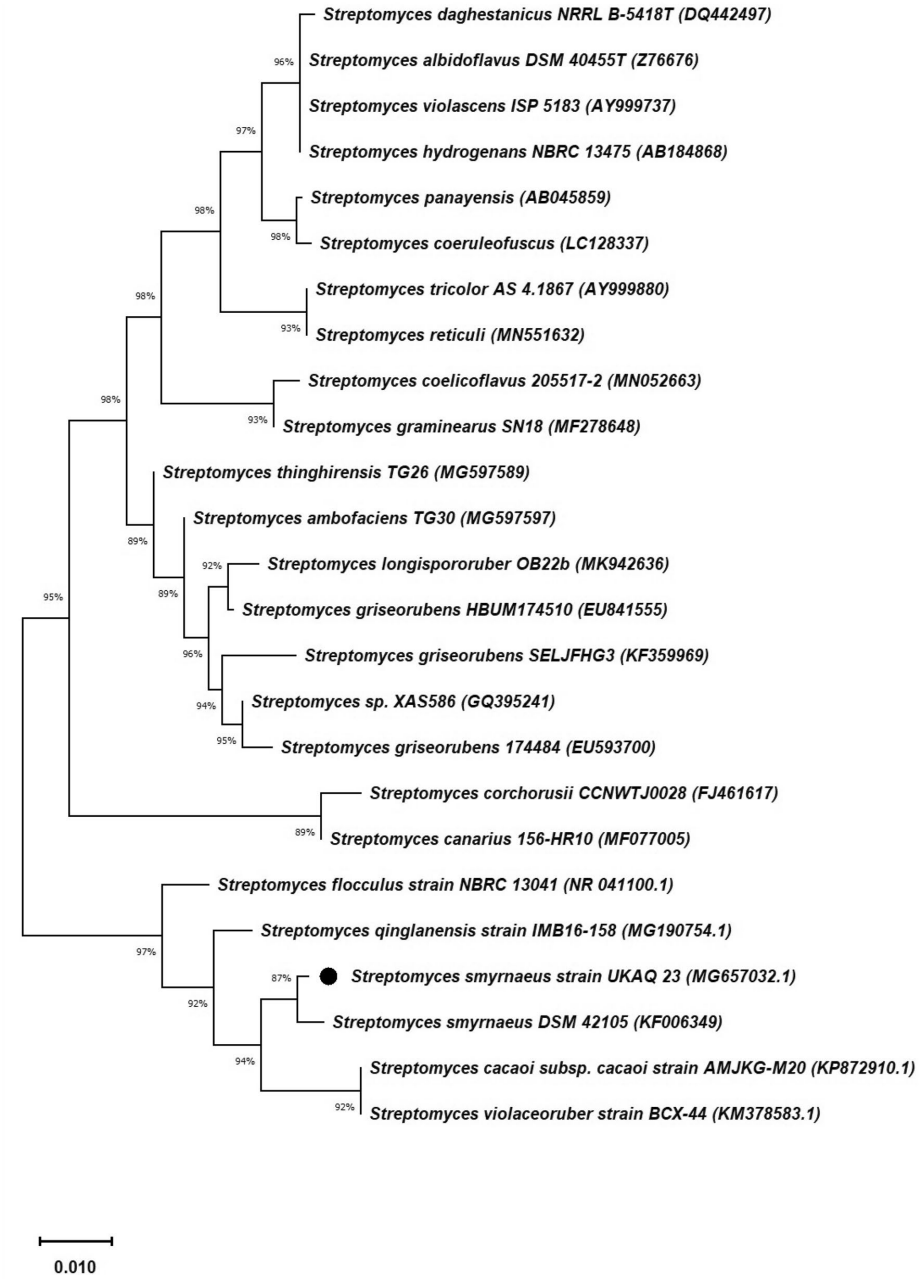
Neighbor-joining (NJ) phylogenetic tree of strain UKAQ_23 based on 16S rRNA gene sequencing generated by MEGA-X. The numbers at branch nodes indicate levels of bootstrap support (%) based on neighbor-joining analysis of 1520 resampled datasets. The NCBI accession numbers are given in parentheses. The bar scale, 0.01, represents the nucleotide substitutions per site.

The 16S rRNA gene sequence of UKAQ_23 was compared with the available sequences in GenBank (NCBI, USA), indicating that UKAQ_23 was closely related to the genus *Streptomyces*. The phylogenetic analysis showed that the strain UKAQ_23 had the highest similarity with *Streptomyces smyrnaeus* DSM 42105 (87%) and *Streptomyces smyrnaeus* (99.47%, NCBI). A neighbor-joining (NJ) phylogenetic tree of UKAQ_23 based on 16S rRNA gene sequencing was generated by MEGA-X (Fig. 2), showing the relationship between UKAQ_23 and the related species belonging to the genus *Streptomyces* that were obtained from the NCBI database. The 16S rRNA gene sequence of strain UKAQ_23 has been submitted to the GenBank (NCBI) with accession number MG657032.1. A culture copy of *Streptomyces smyrnaeus* UKAQ_23 has been deposited at the National Centre for Microbial Resource (NCMR), Pune, MS, India, under the accession number MCC 0192.

**Figure 3:**
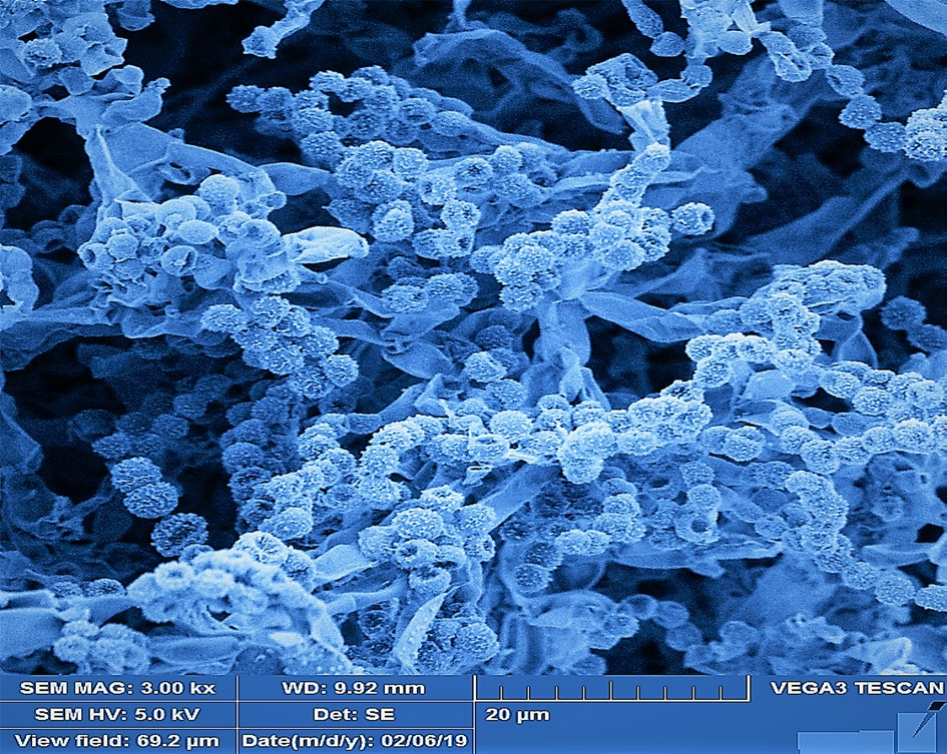
SEM image of *Streptomyces smyrnaeus* UKAQ_23.

The culture characterization of UKAQ_23 revealed that all the tested growth media had supported the growth of UKAQ_23. The color of substrate mycelium ranged from canary-yellow to yellow. The diffusible pigmentation was observed only in GM-5, GM-6, and GM-7, which were yellow, while no diffusible pigmentation was observed in GM-1 to GM-4. The color of aerial mycelium ranged from grey to yellow (see, Supplementary Table S1). The scanning electron microscopy (SEM) image revealed that the spores of UKAQ_23 were smooth and arranged in chains (Fig. 3).

The physiological characterization of UKAQ_23 revealed that the optimum growth of UKAQ_23 was observed in the presence of various carbon sources, including starch maize, starch soluble, sucrose, glycerol, D (−) mannitol, and D ( +) dextrose; nitrogen sources, including ammonium sulfate, glycine, L-asparagine monohydrate, and soybean meal; pH 6.5; temperature 30 °C, and NaCl 0.5% w/v (see, Supplementary Table S2). The biochemical characterization of UKAQ_23 revealed that the isolate was positive for amylase, protease, lipase, nitrate reduction, cellulose decomposition, and 10% skimmed milk coagulation but negative for coagulase, catalase, H_2_S production, and gelatin liquefaction. The isolate was found to be susceptible to chloramphenicol (30 µg) and clindamycin (2 µg), but resistant to amoxycillin (10 µg), ceftriaxone (30 µg), imipenem (10 µg), and tetracycline (30 µg).

### Antibiotic production and optimization of media composition and fermentation conditions

Antibiotic production revealed that ISP-4 broth was the best medium for antibiotic production, with a 17 mm zone of inhibition against the test organism, while other tested fermentation media showed no zone of inhibition against the tested organism. To improve antibiotic production, the ISP-4 medium was further optimized, and it was found that only two of the twenty-four tested fermentation broths, the FM-5, and FM-9, supported antibiotic production, with inhibition zones of 17 mm and 20 mm, respectively, against the tested organism. As a result, FM-5 and FM-9 were selected for further finer optimizations. In submerged fermentation, the antibiotic production was significantly variable; therefore, further optimization of media composition and fermentation conditions was carried out in solid-state fermentation. The highest yield (Mean ± SD) of crude antimicrobial extract from solid-state fermentation was 561.3 ± 0.3 mg/kg fermented agar in a fermentation medium (modified ISP-4 agar) consisting of starch maize 1% w/v, starch soluble 0.6% w/v, (NH_4_)_2_SO_4_ 0.5% w/v, NaCl 0.75% w/v, K_2_HPO_4_ 0.6% w/v, CaCl_2_ 0.15% w/v, MgSO_4_ 0.05% w/v, CaCO_3_ 0.015% w/v, FeSO_4_ 0.010% w/v, ZnSO_4_ 0.005% w/v, agar 1.5% w/v, pH 6.5, inoculum 5% v/w at temperature 35 °C, and an incubation period of 7 days (see, Supplementary Table S3).

### Optimization of antibiotic production by response surface methodology (RSM)

**Figure 4:**
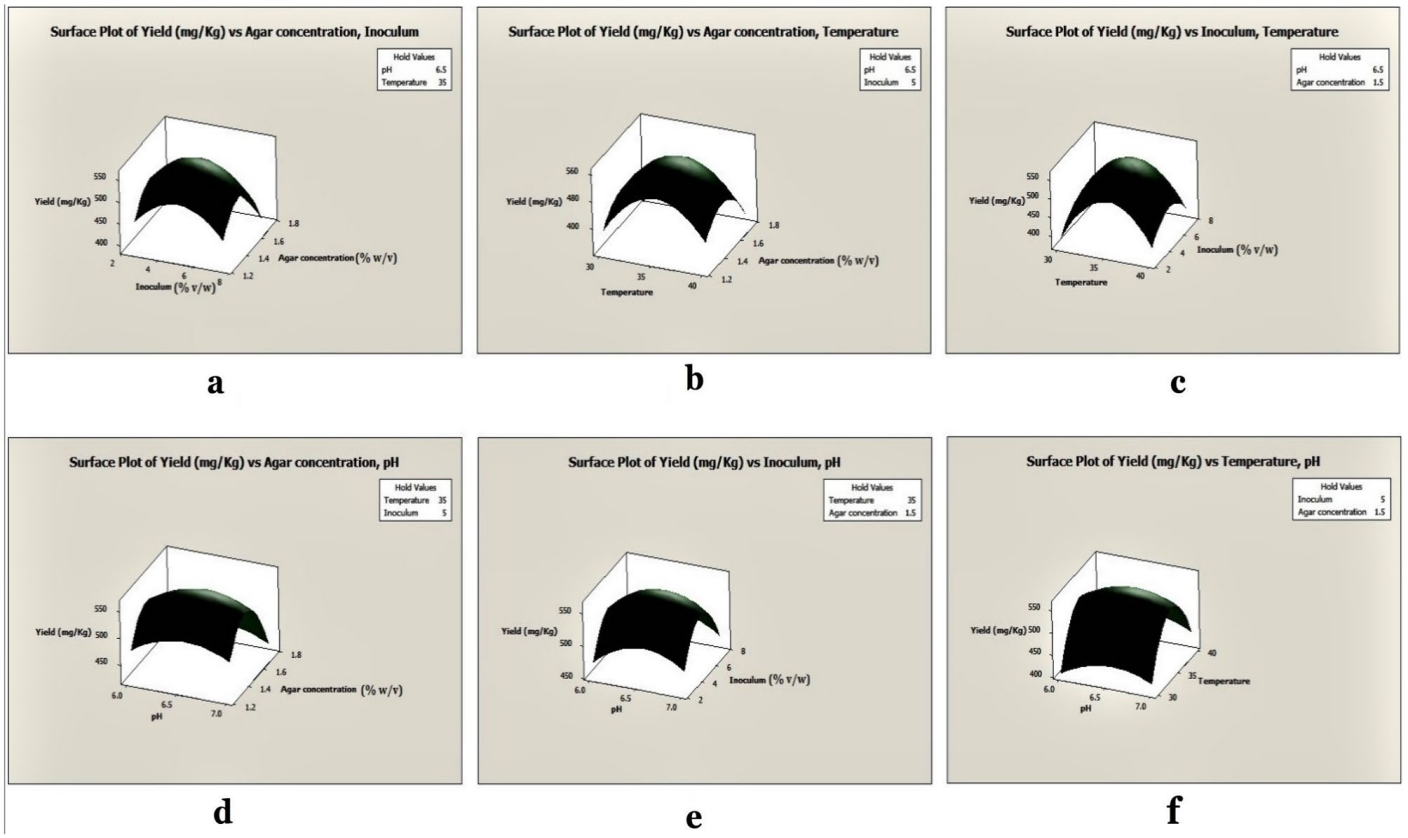
Surface plots of antimicrobial extract yield from UKAQ_23.

**Figure 5:**
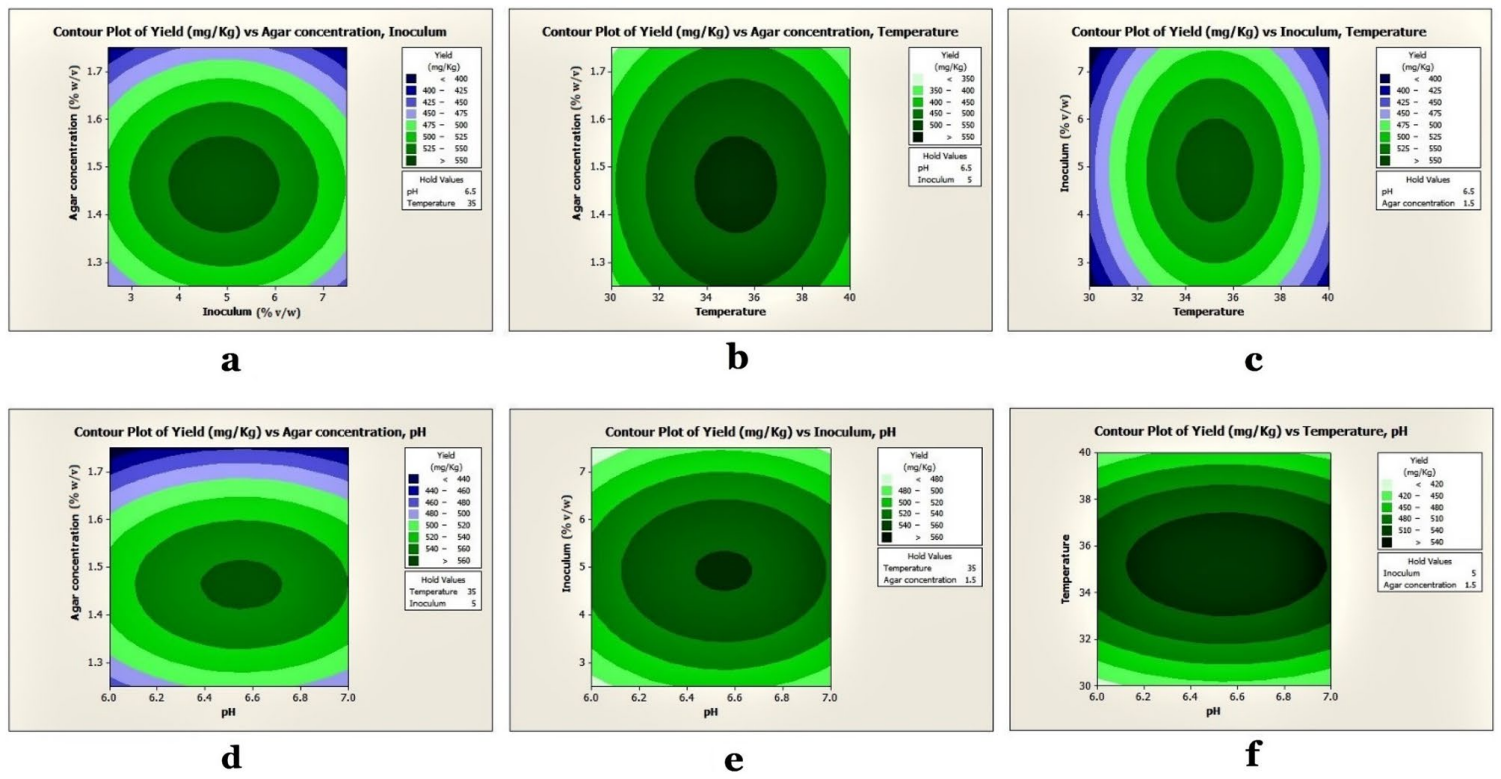
Contour plots of antimicrobial extract yield from UKAQ_23.

The results showed that the linear and quadratic effects of pH, temperature, inoculum volume, and agar concentrations were highly significant (P < 0.05), but the interactive effects of agar concentrations and inoculum volumes were insignificant (P > 0.05). The optimal level of each variable and the effects of their interactions on antibiotic output were explored using three-dimensional (3D) response surface curves (Fig. 4), and the findings were dependent on the strategy of holding one variable constant at its optimum level while the other two variables were varied across the experimental range. The 3D curves of the measured responses indicated interactions between pH, temperature, inoculum volume, and agar concentrations (Figs. 4–5). The crude antimicrobial extract's experimental yield was 561.3 ± 0.3 mg/kg of the fermented agar, whereas the RSM predicted yield of the crude antimicrobial extract was 842.0 ± 0.0 mg/kg of fermented agar (see, Supplementary Table S4). An ANOVA analysis revealed P < 0.05, indicating that the model was significant. The 3D response surface and contour presentations were plotted to investigate the interaction of the different physicochemical factors used and evaluate the optimal range of each factor for maximal antibiotic production.

### Antibiotic extraction and purification

The solid–liquid extraction produced a crude antimicrobial extract that was reddish-orange in color and amorphous. The TLC analysis revealed that the crude antimicrobial extract contained two prominent compounds, K_1_ and K_2_, with Rf values of 0.38 and 0.28, respectively (see, Supplementary Figure S2). The bioautography revealed that both the compounds, K_1,_ and K_2_ had antimicrobial activity against the indicator organism (see, Supplementary Figure S2). Thus, both compounds were selected for further analyses. The comprehensive application of various chromatographic techniques resulted in the isolation of two pure compounds, K_1_ and K_2_. The compounds K_1_ and K_2_ were subjected to further structure elucidation and antimicrobial activity assessments.

### Physicochemical characterization and structure elucidation of compounds K_1_ and K_2_

**Figure 6:**
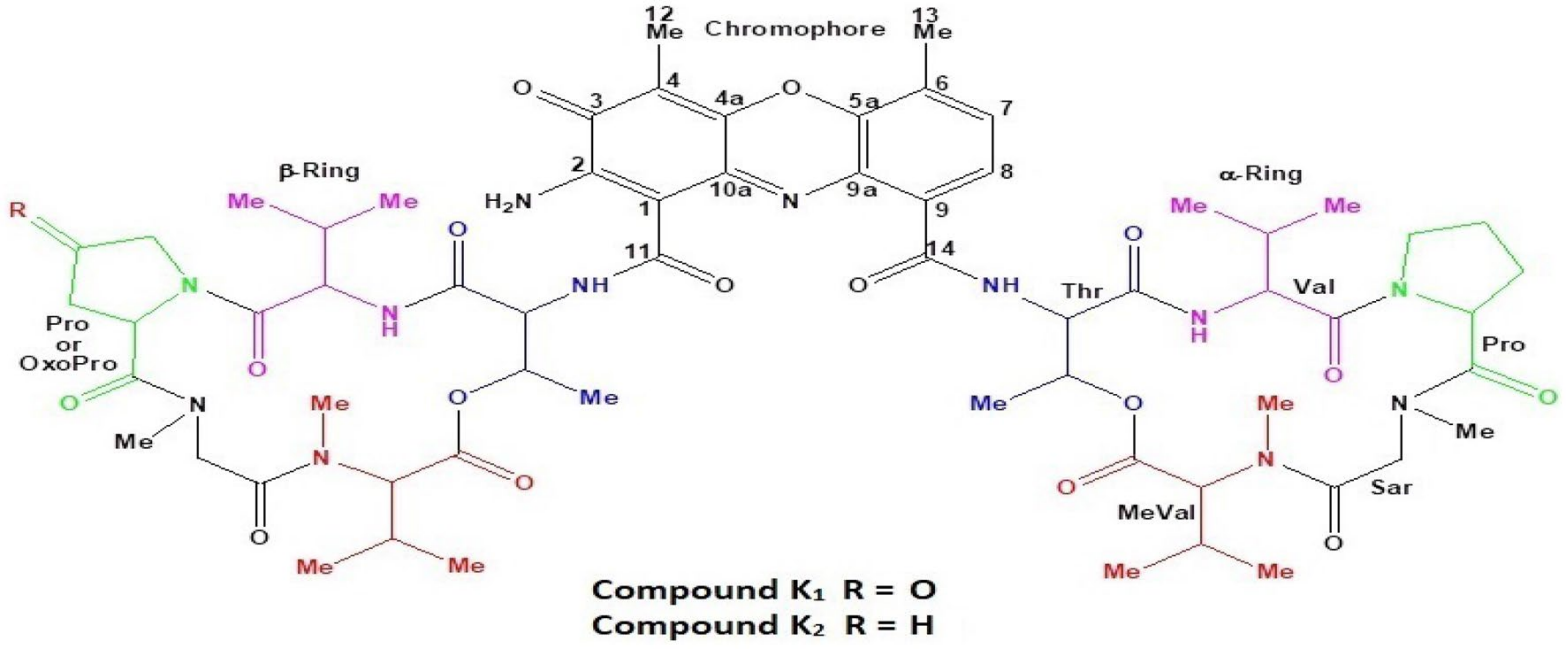
Predicted structure of isolated Actinomycin X_2_ and Actinomycin D.

The purified compounds, K_1,_ and K_2_ based on their physicochemical and spectroscopic analyses, were identified as actinomycin X_2_ and actinomycin D, respectively (Fig. 6), (Table 1).

**Table 1:** Physicochemical characteristics of the isolated compounds K_1_ and K_2_.

Physicochemical characteristics	Isolated compounds	
	K_1_	K_2_
Color and appearance	Reddish-orange amorphous	Reddish-orange amorphous
Solubility	**Highly soluble:** chloroform, ethyl acetate, dichloromethane, acetonitrile, methanol, acetone **Partial-soluble:** ethanol, dimethylformamide, dimethyl sulphoxide, water **Non-soluble:** n-hexane	**Highly soluble:** chloroform, ethyl acetate, dichloromethane, acetonitrile, methanol, acetone **Partial-soluble:** ethanol, dimethylformamide, dimethyl sulphoxide, water **Non-soluble:** n-hexane
Melting point (°C)	245–250 with decomposition	245–250 with decomposition
UV–Visible (λ_max_, nm)	239, 440 (Methanol)	215, 442 (Methanol)
FT-IR (υ_max_, cm^-1^)	1583 (N–H bending) 1648–1668 (C=C stretching) 1705–1740 (C=O stretching) 2849–2916 (C–H stretching)	1587 (N–H bending) 1627–1656 (C=C stretching) 1728 (C=O stretching) 2853–2957 (C–H stretching)
( +)-HR-ESI-MS	*m/z* 1269.6170 [M + H]^+^ (calcd for C_62_H_85_N_12_O_17_, 1269.616); *m/z* 1291.5997 [M + Na]^+^ (calcd for C_62_H_84_N_12_O_17_Na, 1291.598)	*m/z* 1255.638 [M + H]^+^ (calcd. for C_62_H_87_N_12_O_16_, 1255.637); 1277.623 [M + Na]^+^ (calcd. for C_62_H_86_N_12_O_16_Na, 1277.619)
Monoisotopic masses	1268.617 g/mol	1254.638 g/mol

The compounds, K_1_ and K_2_, were isolated as reddish-orange amorphous powders. Their UV–Visible spectra showed absorption maxima (λ_max_) at 239 and 440 nm for the compound K_1_ (see, Supplementary Figure S3**)** and 215 and 442 nm for the compound K_2_ (see, Supplementary Figure S4), corresponding to the known actinomycins, X_2_ and D^17^. The IR spectra of K_1_ and K_2_ indicated the presence of amide, ketone, and aromatic ring functionalities (see, Supplementary Figure S5-S6).

#### Mass spectrometry

The HR-ESI–MS spectrum of K_1_ showed a molecular ion peak at *m/z* 1269.6170 [M + H]^+^ and 1291.5997 [M + Na]^+^, which corresponded to Actinomycin X_2_ (see, Supplementary Figure S7). The mass ion peak at *m/z* 1269.6170 [M + H]^+^ corresponded to the molecular formula, C_62_H_85_N_12_O_17_ [M + H]^+^ of product K_1_. The molecular formula was consistent with the Actinomycin X_2_. The analysis of the MS^2^ spectrum of K_1_ showed two types of fragments (see, Supplementary Table S5); fragment identical to Actinomycin D^17^ with molecular mass: *m/z* 300.192 Da, and another fragment with molecular mass: *m/z* 314.171 Da, an increment of ~ 14 Da mass unit for Actinomycin D. According to the published data, these fragments corresponded to sequence [Pro-Sar-Me-Val-OH + H] ^+^ and [OxoPro-Sar-Me-Val-OH + H] ^+^ , respectively. The fragments at *m/z* 300.192 and 314.171 obtained by MS^2^ of the ions at *m/z* 635.3125 ([M + 2H]^2+^) were subjected to MS^3^. MS^3^ spectrum demonstrated the elimination of Me-Val residue, which is in good agreement with the proposed sequence. On the other hand, a neutral loss of 81 Da, which was interpreted as eliminating the OxoPro fragment with the molecular formula, C_4_H_3_NO, and fits into the proposed structural formula. The MS^3^ spectrum demonstrated that compound K_1_ contains Oxo-Pro moiety instead of Pro in one chain (see, Supplementary Figure S7). A literature search revealed that the actinomycin analog with OxoPro in one of the peptide rings has been reported, *i.e.*, Actinomycin X_2_.

An LC–MS comparison between compound K_1_ and standard Actinomycin X_2_ revealed similar retention times (see, Supplementary Figure S8) and MS (see, Supplementary Figure S9), whereas an LC–MS-MS comparison revealed similar fragmentation patterns (see, Supplementary Figure S10). These experiments demonstrated that there was no difference between the compared compounds. As a result, it was concluded that compound K_1_ is similar to Actinomycin X_2_.

The HR-ESI–MS spectrum of K_2_ showed molecular ion peaks at *m/z* 1255.638 [M + H]^+^ and 1277.623 [M + Na]^+^, which corresponded to Actinomycin D (see, Supplementary Figure S11). These molecular ion peaks corresponded to the molecular formula C_62_H_87_N_12_O_16_ [M + H]^+^ of K_2_. The mass and molecular formula were consistent with Actinomycin D. The CID MS/MS fragmentation spectrum of compound K_2_ was compared with the standard Actinomycin D^17^ (see, Supplementary Table S6).

An LC–MS comparison of compound K_2_ with the standard Actinomycin D revealed a similar retention time (see, Supplementary Figure S12) and same MS (see, Supplementary Figure S13), whereas an LC–MS-MS comparison revealed a similar fragmentation pattern (see, Supplementary Figure S14). These observations revealed that there was no difference between the compared compounds. As a result, it was concluded that compound K_2_ is similar to Actinomycin D.

#### NMR spectroscopy

The Nuclear Magnetic Resonance (NMR) is a powerful analytical tool that has been used as a gold standard for molecular identification^18–20^, structural elucidation, and drug discovery^20–22^. In this study, several 1D and 2D NMR techniques were used for the structure elucidation of the isolated compounds. The NMR spectroscopic data for the K_1_ peptide displayed typical characteristics of an actinomycin molecule, including the presence of a phenoxazinone unit and two pentapeptide lactone rings. The ^1^H NMR spectrum of K_1_ showed two *ortho*-coupled aromatic protons at δ 6.66 (d, *J* = 7.7 Hz, H-8) and 6.40 (d, *J* = 7.7 Hz, H-7) together with two methyl protons at δ 2.59 (s, H_3_-13) and δ 2.25 (s, H_3_-12), characteristic for the phenoxazinone chromophore (see, Supplementary Table S7). Furthermore, the ^1^H NMR spectrum of K_1_ displayed characteristics of a typical peptide, with NH protons at δ 7.22–8.21, 10 amino acid α-protons at δ 2.68–6.62, and two ester carbinol protons at δ 5.20 (qd, *J* = 6.3, 2.7 Hz, H-3, Thr, α-ring) and 5.29 (qd, *J* = 6.2, 2.7 Hz, H-3, Thr, β-ring), four *N-*Me singlets at δ 2.94 (s, H_3_-Val, β-ring), δ 2.93 (s, H_3_-Val, α-ring), δ 2.89 (s, H_3_-Sar, β-ring), and δ 2.88 (s, H_3_-Sar, α-ring), as well as 10 additional methyl doublets at δ 1.30/δ 1.17 (d, *J* = 6.5/6.3 Hz, H_3_-4, Thr), δ 1.19/δ 1.15 (d, *J* = 6.7/6.7 Hz, H_3_-4, Val), δ 0.95/δ 0.94 (d, *J* = 7.1/7.1 Hz, H_3_-5, Val), δ 1.03/δ 1.00 (d, *J* = 6.0/5.9 Hz, H_3_-4, Me-Val) and δ 0.79/δ 0.78 (d, *J* = 6.5/7.6 Hz, H_3_-5, Me-Val). These characteristics signals were accounted for two pentapeptide lactone rings. The ^13^C NMR and DEPT-135 spectra revealed the presence of 10 amino acid residues from 10 α-amino acid carbon signals at δ 50.72–70.91 and the 10 carbonyl signals at δ 165.31–173.47, indicating that K_1_ is a peptide (see, Supplementary Table S7). The ^13^C NMR and DEPT-135 spectra also showed 16 methyl groups from which two were assigned to C-12 (δ 7.20) and C-13 (δ 14.48) of the phenoxazinone, 5 methylenes from which three were assigned to C-3, C-4, and C-5 of the α-ring proline, and two for the C-3 and C-5 of β-ring proline, indicating that one of the methylene carbon of this proline was modified, a 2 sp^2^ methines (C-7 and C-8 of phenoxazinone nucleus), 6 sp3 methines including two oxygenated (C-3 of each threonine), and four non-oxygenated (C-3 of each valine and methyl-valine), and 12 sp^2^ quaternary carbons including three carbonyls for C-3, C-11, and C-14 of the phenoxazinone nucleus. The ongoing data are in agreement with spectral features observed for the actinomycins. Additionally, the downfield carbonyl signal at δ 208.27 was assigned to C-4 of β-ring proline, indicating that compound K_1_ has dissimilar pentapeptide lactone rings, one ring contains proline, and the other contains oxoproline. After an extensive 2D-NMR (HSQC, COSY, and HMBC) analyses, the amino acid residues in each peptide ring were identified as Thr, Val, Pro, Sar, and Me-Val in α-ring, and Thr, Val, OxoPro, Sar, and Me-Val in β-ring (see, Supplementary Table S7). The amino acid α-protons showed correlations with the corresponding amino acid’s α-carbons as shown in the HSQC spectrum of K_1_. Furthermore, the five amino-acids residues in both the pentapeptide lactone rings were identified from their unique spin system, as shown in the COSY spectrum^23^. The sequence of amino acids in these two peptide lactone units in K_1_ was identical to those of the Actinomycin X_2_. The sequence of amino acids residue in α-ring was deduced from the following HMBC correlations: H-3 (δ 5.29) of Thr with C-1 (δ 165.83) of Me-Val; H-2 (δ 2.68) and N-Me (δ 2.94) of Me-Val with C-1 (δ 165.31) of Sar; H_2_-2 (δ 4.75 and 3.69) and N-Me (δ 2.88) of Sar with C-1 (δ 172.57) of Pro; H-2 (δ 6.02) of Pro with C-1 (δ 173.03) of Val and H-2 (δ 3.72) and NH (δ 8.21) of Val with C-1 (δ 168.32) of Thr. The location of the α-ring pentapeptide lactone at C-14 of the phenoxazinone unit was confirmed by the long-rang correlation between H-2 (δ 4.52) of Thr and C-14 (δ 165.31) of the phenoxazinone part. Similarly, the sequence of amino acids residue in β-ring was deduced from the HMBC correlations as H-3 (δ 5.20) of Thr with C-1 (δ 166.88) of Me-Val; H-2 (δ 2.73) and N-Me (δ 2.93) of Me-Val with C-1 (δ 165.71) of Sar; H_2_-2 (δ 4.64 and 3.68) and N-Me (δ 2.89) of Sar with C-1 (δ 172.13) of OxoPro; H-2 (δ 6.62) of OxoPro with C-1 (δ 173.47) of Val and H-2 (δ 3.61) and NH (δ 7.68) of Val with C-1 (δ 167.99) of Thr. The location of the β-ring pentapeptide lactone at C-11 of the phenoxazinone part was confirmed by the long-rang correlations between H-2 (δ 4.58) of Thr and C-11 (δ 166.92) of the phenoxazinone chromophore. Additionally, the phenoxazinone chromophore was established from the ongoing 2D correlations: H-7 and H-8 were correlated with the carbons at δ 129.70 (C-8) and δ 125.65 (C-7) in the HSQC spectrum and were also correlated to each other in the COSY spectrum. In the HMBC spectrum, H-7 showed cross-peaks with C-5a (δ 139.93), C-9 (δ 131.59) and C-13 (δ 14.48), whereas the H-8 showed cross-peaks with C-6 (δ 127.20), C-9a (δ 128.60) and C-14 (δ 165.31). Furthermore, the methyl protons at δ 2.59 (H_3_-13) correlated with C-5a (δ 139.93), C-6 (δ 127.20) and C-7 (δ 129.70) and the methyl protons at δ 2.29 (H_3_-12) correlated with C-3 (δ 178.53), C-4 (δ 113.00), and C-4a (δ 144.46) protons. Based on the NOESY spectrum and their similar NMR pattern, the relative configuration of compound K_1_ was assigned to be identical to that of Actinomycin X_2_ (see, Supplementary Figure S15-S21). The compound K_1_ was identified as Actinomycin X_2_ based on ongoing data and comparison with previously published spectroscopic and chromatographic data^17,23–28^.

### Antimicrobial activity of compounds K_1_ and K_2_

#### Primary antimicrobial activity

**Figure 7:**
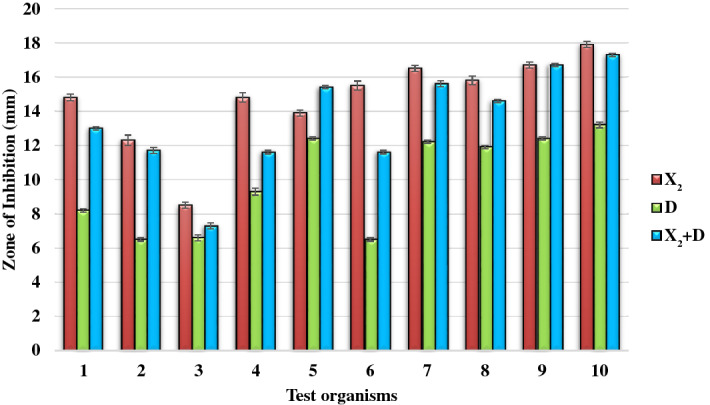
Primary antimicrobial screening of isolated actinomycins X_2_ and D.

**Figure 8:**
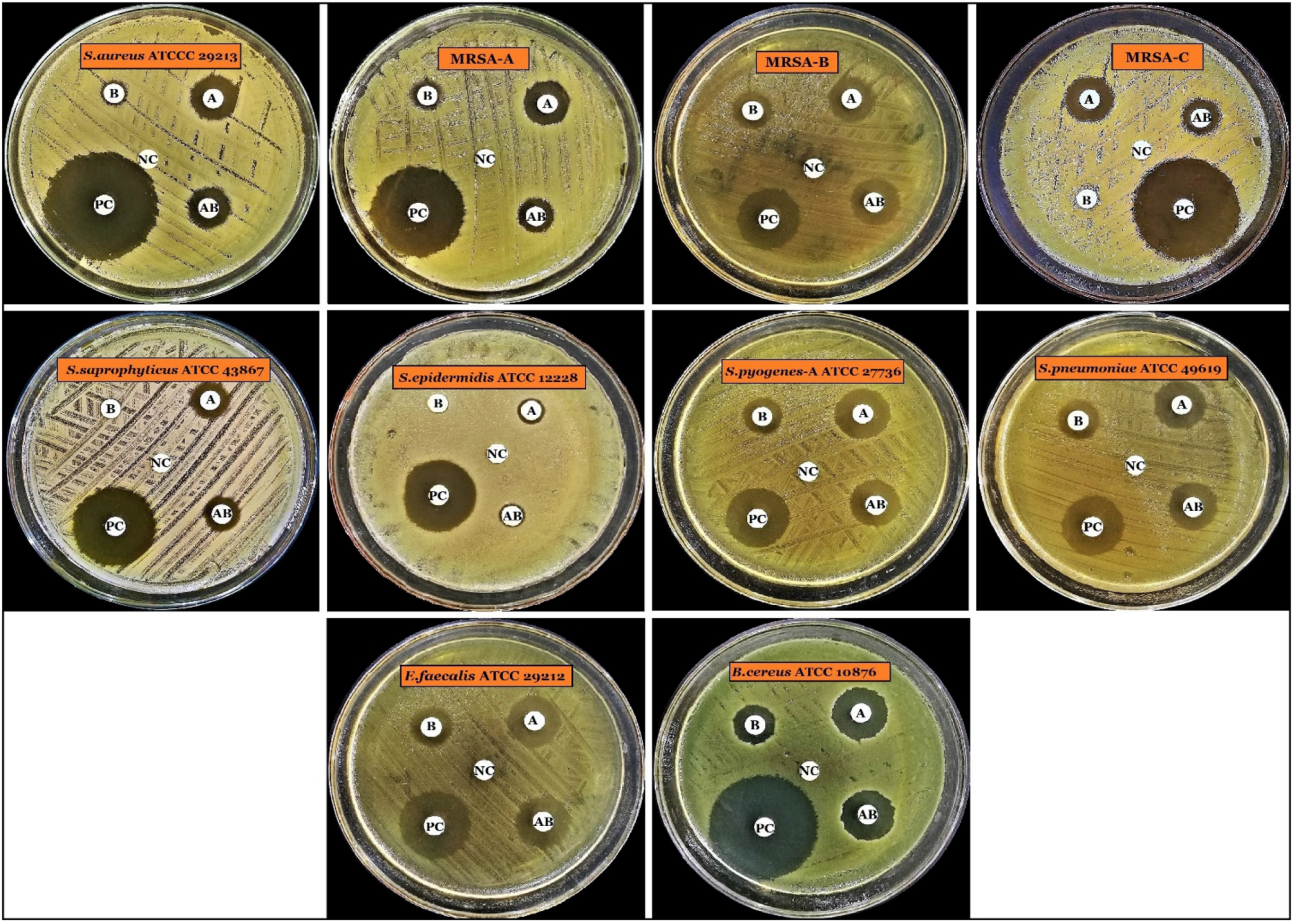
Primary antimicrobial activity of isolated actinomycins X_2_ and D. A = Actinomycin X_2_ (5 µg/disc), B = Actinomycin D (5 µg/disc), AB = Actinomycins-complex (X_2_ + D; 1:1 w/w; 5 µg/disc), *PC* Positive control/Levofloxacin (5 µg/disc), *NC* Negative control/Methanol (20 µl/disc).

The primary antimicrobial activity of the compounds K_1_ and K_2_ demonstrated that Actinomycin X_2_ had a larger zone of inhibition, *i.e.*,14.7 ± 2.7 mm and Actinomycin D had a smaller zone of inhibition, *i.e.*, 9.9 ± 2.8 mm, whereas actinomycins-mixture (X_2_ + D, 1:1 w/w) had a medium zone of inhibition, *i.e.*, 13.5 ± 3.0 mm (Mean ± SD), against the Gram-positive test bacteria, at a concentration of 5 µg/disc. According to the results, Actinomycin X_2_ was the most potent antibiotic, while Actinomycin D was the least potent, and actinomycins-mixture showed mild potency. Levofloxacin (control antibiotic) inhibited non-MRSA bacteria with a zone of inhibition of 25.6 ± 6.91 mm and MRSA bacteria with a zone of inhibition of 28.8 ± 7.14 mm (Mean ± SD) (Figs. 7, 8) (see, Supplementary Table S8). Based on the above findings, Actinomycin X_2_ and Actinomycin D were selected for further investigation, and actinomycins-complex (X_2_ + D) was omitted.

#### Secondary antimicrobial activity (MIC and MBC)

MIC of Actinomycin X_2_ ranged from 1.56 to 12.5 μg/ml for non-MRSA test bacteria and from 3.125 to 12.5 μg/ml for MRSA test bacteria, while MIC of the isolated Actinomycin D ranged from 3.125 to 12.5 μg/ml for non-MRSA test bacteria and from 12.5 to 25 μg/ml for MRSA test bacteria. Levofloxacin (control antibiotic) inhibited the growth of all the test organisms at a concentration of 5 µg/ml (Table 2). MBC of Actinomycin X_2_ ranged from 6.25 to 50 μg/ml for non-MRSA test bacteria and from 12.5 to 50 μg/ml for MRSA test bacteria, while MBC of Actinomycin D ranged from 25 to 50 μg/ml for non-MRSA test bacteria and 50 μg/ml for MRSA test bacteria (Table 2).

**Table 2:** MIC and MBC of isolated actinomycins X_2_ and D.

Test organisms	MIC (µg/ml)		MBC (µg/ml)		Levofloxacin (5 µg/ml)
	Actinomycin X_2_	Actinomycin D	Actinomycin X_2_	Actinomycin D	
**Non-MRSA bacteria**					
*S.aureus* ATCC 29213	1.56	6.25	6.25	25	Inhibition
*S.saptophyticus* ATCC 43867	3.125	12.5	12.5	50	Inhibition
*S.epidermidis* ATCC 12228	1.56	6.25	6.25	25	Inhibition
*S.pyogenes*-A ATCC 27736	12.5	12.5	25	50	Inhibition
*S.pneumoniae* ATCC 49619	12.5	12.5	25	50	Inhibition
*E.faecalis* ATCC 29212	12.5	12.5	50	50	Inhibition
*B.cereus* ATCC 10876	1.56	3.125	12.5	25	Inhibition
MRSA bacteria*					
MRSA-A	3.125	25	12.5	50	Inhibition
MRSA-B	12.5	25	50	50	Inhibition
MRSA-C	3.125	12.5	12.5	50	Inhibition

*Clinical isolates.

### Statistical analyses

The statistical analyses revealed that; there was a statistically significant difference (P < 0.05) between groups of tested bacterial strains as determined by One-way ANOVA, *i.e.*, for Actinomycin X_2_; F (9,20) = 436.605, p = 0.000, for Actinomycin D; F (9,20) = 1371.294, p = 0.000, and for actinomycins-mixture; F (9,20) = 1730.333, p = 0.000 (Table 3); there was a statistically significant difference (P < 0.05) between tested actinomycins (X_2_, D, X_2_ + D) as determined by One-way ANOVA, *i.e.*, for Actinomycin X_2_; F (9) = 397.826, p = 0.000, for Actinomycin D; F (9) = 1109.126, p = 0.000, and for actinomycin-mixture; F (9,20) = 1018.509, p = 0.000 (Table 4).

**Table 3:** One-way ANOVA for primary antimicrobial activity of isolated actinomycins X_2_, D, and X_2_ + D.

ANOVA						
		Sum of Squares	df	Mean Square	F	Sig
Actinomycin X_2_	Between groups	192.543	9	21.394	436.605	.000
	Within groups	.980	20	.049		
	Total	193.523	29			
Actinomycin D	Between groups	209.808	9	23.312	1371.294	.000
	Within groups	.340	20	.017		
	Total	210.148	29			
Actinomycin-mixture (X_2_ + D) (1:1, w/w)	Between groups	249.168	9	27.685	1730.333	.000
	Within groups	.320	20	.016		
	Total	249.488	29

**Table 4:** One-way ANOVA for primary antimicrobial activity of isolated actinomycins X_2_, D, and X_2_ + D.

Robust tests of equality of means					
		Statistic^a^	df1	df2	Sig
Actinomycin X_2_	Welch	397.826	9	8.133	.000
Actinomycin D	Welch	1109.126	9	8.132	.000
Actinomycin-mixture (X_2_ + D) (1:1, w/w)	Welch	1018.509	9	8.134	.000

^a^Asymptotically F distributed.

### Molecular docking of isolated actinomycin X_2_ (K_1_) and actinomycin D (K_2_)

**Figure 9:**
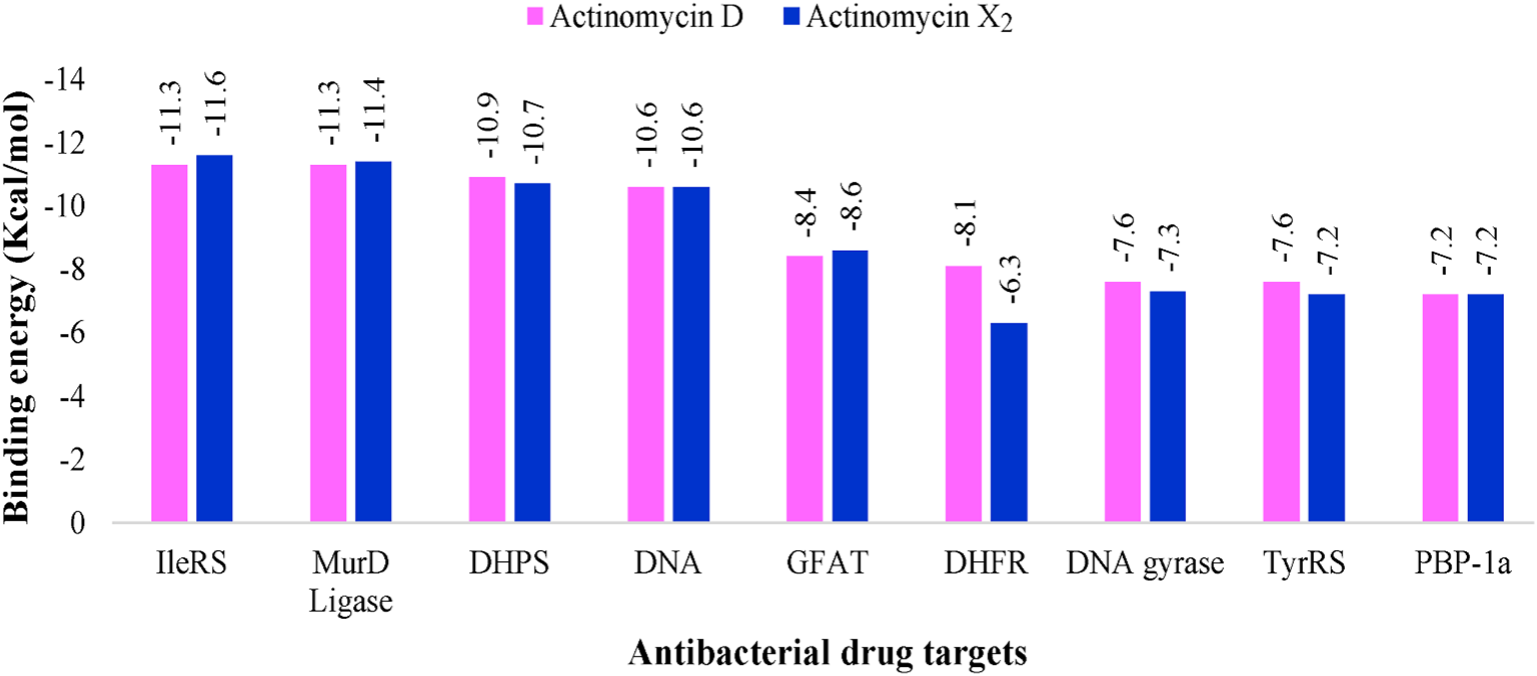
The plot of molecular docking predicted binding energy (Kcal/mol) and several antibacterial drug targets. *IleRS* isoleucyl-tRNA synthetase, *MurD* UDP-N-acetylmuramoyl-L-alanine:D-glutamate, *DHPS* dihydropteroate synthase, *DNA* deoxyribonucleic acid, *GFAT* glucosamine-fructose-6-phosphate aminotransferase, *DHFR* dihydrofolate reductase, *TyrRS* tyrosyl-tRNA synthetase, *PBP-1a* penicillin-binding protein 1a.

The in-silico molecular docking experiments predicted affinity in terms of binding energies of the compounds K_1_ and K_2_ against the DNA and eight other potential antibacterial protein targets (Table 5). The molecular docking interactions are represented in Fig. 9. Detailed in-silico intermolecular interaction analysis is presented in Table 6. Both compounds, K_1_ and K_2,_ exhibited maximum affinity with isoleucyl-tRNA synthetase showing binding energy of − 11.6 kcal/mol and − 11.3 kcal/mol, respectively. However, penicillin-binding protein-1a was observed as the least favorable target for both docked compounds demonstrating an equal value of binding energy as − 7.2 kcal/mol; interestingly, both compounds preferred to bind with DNA, indicating equipotent in terms of docking predicted affinity of − 10.6 kcal/mol. In general, a very subtle difference was noted between the binding energies of compounds K_1_ and K_2_ except dihydrofolate reductase target where compound K_2_ (− 8.1 kcal/mol) was observed as more potent than K_1_ (− 6.3 kcal/mol) with an appreciable difference in the binding energy (Table 5).

**Table 5:** Results of molecular docking of compounds K_1_ and K_2_.

Target	PDB ID	Reference	Docking predicted binding energy (Kcal/mol)	
			K_1_	K_2_
DNA	1MNV	^29^	− 10.6	− 10.6
DNA gyrase	3TTZ	^30,31^	− 7.3	− 7.6
Dihydropteroate synthase	2VEG	^30,32^	− 10.7	− 10.9
Dihydrofolate Reductase	3SRW	^30,33^	− 6.3	− 8.1
Glucosamine-fructose-6-phosphate aminotransferase	2VF5	^34,35^	− 8.6	− 8.4
Isoleucyl-tRNA synthetase	1JZQ	^30,36^	− 11.6	− 11.3
Tyrosyl-tRNA synthetase	1JIJ	^37,38^	− 7.2	− 7.6
Penicillin-binding protein 1a	3UDI	^30,39^	− 7.2	− 7.2
UDP-N-acetylmuramoyl-L-alanine:D-glutamate (MurD) ligase	2X5O	^40,41^	− 11.4	− 11.3

**Table 6:** Intermolecular interactions observed between docked compounds K_1_ and K_2_ and drug targets. Bond distances are given in parentheses.

Targets	Actinomycin X_2_ (K_1_)		Actinomycin D (K_2_)	
	Residues involved in H-bonding	Residues involved in hydrophobic interactions	Residues involved in H-bonding	Residues involved in hydrophobic interactions
DNA	A:DT2 (3.02)	A:DG3 (4.23)	B:DG15 (2.52)	A:DG3 (4.31)
	A:DG3 (2.52)	B:DG15 (3.61)	B:DC16 (2.46)	B:DG15 (3.63)
	A:DG3 (2.44)	B:DC16 (4.56)	A:DG3 (2.51)	B:DC16 (4.49)
	A:DC4 (2.77)	A:DG3 (3.58)	A:DG3 (2.51)	A:DG3 (3.58)
	A:DT5 (2.58)	A:DC4 (5.52)	B:DG15 (1.81)	A:DC4 (5.57)
	B:DG15 (1.96)	B:DG15 (3.66)	B:DT14 (2.92)	B:DG15 (3.59)
	B:DG15 (2.58)	A:DG3 (4.20)	B:DG15 (2.39)	A:DG3 (4.14)
	B:DC16 (2.47)	A:DC4 (4.09)		A: DC4 (4.06)
	B: DA17 (2.92)	B: DG15 (5.11)		B: DG15 (4.99)
		A: DG3 (5.59)		A: DG3 (5.69)
		A: DG3 (4.27)		A: DG3 (4.29)
		A: DG3 (3.86)		A: DG3 (3.78)
		B: DG15 (3.82)		B: DG15 (4.80)
		B: DG15 (4.91)		B: DG15 (4.35)
		A: DG3 (4.31)		A: DG3 (5.27)
		A: DG3 (5.12)		A: DG3 (4.09)
		A: DC4 (3.85)		A: DC4 (3.85)
		B: DG15 (3.95)		B: DG15 (3.95)
		B: DG15 (4.21)		B: DG15 (4.24)
		B: DG15 (5.33)		B: DG15 (5.28)
DNA gyrase	Asp57 (3.00)	Arg84 (4.10)	Asp57 (2.47)	Glu58 (5.53)
	Asp57 (2.22)	Glu58 (4.39)	Gln91 (2.85)	Arg84 (4.14)
		Ala61 (3.92)		Glu58 (4.27)
		Ala61 (3.71)		Ala61 (3.89)
		Ile86 (4.38)		Ala61 (3.64)
		Val101 (5.08)		Ile86 (4.58)
		Pro87 (4.77)		Ile86 (4.47)
		Ile102 (5.46)		Val101 (5.29)
		Ile102 (4.47)		Pro87 (4.73)
				Ile86 (5.42)
				Ile102 (5.42)
				Ile102 (4.40)
Dihydropteroate synthase	Gln38 (2.44)	Pro152 (3.97)	Gln38 (2.41)	Pro152 (4.22)
	Arg236 (2.25)	Pro152 (4.18)	Arg236 (2.24)	Pro152 (4.40)
	Lys237 (2.85)	Lys237 (4.76)	Lys237 (2.22)	Lys237 (4.74)
	Arg282 (2.71)	Arg236 (5.29)	Arg282 (2.64)	Arg236 (5.23)
	Arg282 (2.75)	Arg236 (4.69)	Arg282 (2.77)	Arg236 (4.82)
	Gln38 (2.14)	Phe154 (4.93)	Gln38 (2.17)	Phe154 (4.93)
Dihydrofolate Reductase	Leu29 (2.54)	Leu29 (4.76)	Leu21 (2.25)	Lys53 (3.95)
		Leu55 (5.16)	Lys33 (2.08)	Lys53 (4.47)
		Lys30 (4.24)	Arg58 (2.81)	Pro56 (4.88)
		His24 (4.93)	His24 (2.54)	Lys33 (4.78)
		His24 (4.27)	Ile51 (2.39)	Pro56 (4.06)
				Lys53 (4.85)
				Lys53 (4.81)
				His24 (5.12)
Glucosamine-fructose-6-phosphate aminotransferase	Thr352 (2.55)	Val399 (5.13)	Thr352 (2.75)	Val399 (5.24)
	Val605 (2.54)	Ile326 (5.49)	Val605 (2.30)	Cys300 (5.26)
		Cys300 (5.17)		Val605 (4.29)
		Val605 (4.38)		Leu601 (4.27)
		Leu601 (4.19)		Tyr304 (5.34)
		Tyr304 (5.34)		
Isoleucyl-tRNA synthetase	Arg391 (2.56)	Asp553 (4.49)	Gln554 (2.30)	His54 (3.95)
	His581 (3.07)	Asp553 (4.38)	Met592 (2.71)	His54 (4.43)
	Asn50 (3.19)	His54 (4.68)	Ser593 (2.05)	His54 (5.82)
	Leu52 (3.29)	Leu195 (4.75)	Asp553 (2.72)	Lys591 (4.31)
	Gly551 (2.77)	Leu583 (5.00)		Val599 (3.74)
	Ser593 (3.09)	Ile584 (3.88)		Pro90 (4.58)
		Leu583 (5.48)		Val599 (5.28)
		His581 (4.49)		His581 (4.87)
Tyrosyl-tRNA synthetase	-	Lys84 (5.08)	Thr42 (2.98)	Glu86 (4.64)
		Leu223 (4.29)	Lys231 (3.05)	Lys84 (5.10)
		Lys231 (5.42)		Leu223 (5.06)
				Lys231 (4.63)
				Ala239 (4.35)
				Lys84 (5.07)
Penicillin-binding protein 1a	Ser470 (2.28)	Tyr707 (4.98)	Ser470 (2.31)	Asp471 (5.48)
	Arg488 (2.56)		Arg473 (2.71)	Arg705 (4.14)
	Tyr707 (2.83)		Arg488 (2.45)	Tyr707 (4.98)
UDP-N-acetylmuramoyl-L-alanine:D-glutamate (MurD) ligase	Gly73 (2.89)	Arg37 (4.85)	Gly73 (2.83)	Arg37 (4.92)
	Asp185 (2.63)	Lys348 (4.27)	Asp185 (2.77)	Lys348 (4.31)
	Asn138 (2.82)	Leu416 (5.23)	Asn138 (2.81)	Leu416 (5.29)
	Gly73 (3.04)	Leu416 (5.32)	Gly73 (3.05)	Leu416 (5.49)
	Arg186 (2.73)	Pro72 (4.91)	Arg186 (2.72)	Leu416 (5.09)
		Arg37 (4.12)		Arg37 (4.10)
		Arg37 (5.26)		Arg37 (5.27)
		Leu416 (5.00)		Leu416 (4.97)
				Phe422 (5.47)

## Discussion

The rising prevalence of antibiotic-resistant bacteria hampers the efficacy of the currently available antibiotics and the therapeutic efficiency. This highlights an urgent need to search for new antibiotics with diverse antimicrobial activity to treat the pathogens that are resistant to conventional antibiotics. Ample evidence suggests that terrestrial actinomycetes are still the most promising candidates for discovering new bioactive compounds active against a wide range of pathogenic microorganisms. The isolation of biologically active compounds from terrestrial actinomycetes is still a field of interest in the quest for bioactive compounds^42–46^.

Previously, we isolated *Bacillus pumilus* strains from soil samples collected from Unaizah, Saudi Arabia, and found that the isolated strains were bioactive against various human pathogens, including Gram-positive and Gram-negative test bacteria^3,4,8^. These recent studies motivated us to isolate antibiotic-producing soil organisms from the mangrove sediment samples of Jubail, Saudi Arabia. In the present study, twenty-five mangrove sediment samples were collected from Jubail, Saudi Arabia, and then screened for the existence of bioactive actinomycetes strains. Only one strain, *Streptomyces smyrnaeus* UKAQ_23, exhibited extremely potent antimicrobial activity among the five actinomycetes strains isolated from the twenty-five collected mangrove sediment samples. The selected isolate was employed for antibiotic production in a wide range of fermentation media, resulting in antibiotic production in an inorganic salt medium (ISP-4). The media composition and fermentation conditions were optimized to achieve maximum antibiotic production, resulting in high yield antibiotic production in modified inorganic salt medium (modified ISP-4 agar) by solid-state fermentation. The antibiotics were extracted using a solid–liquid extraction method, and the purification was performed using various chromatographic techniques, resulting in the isolation of two purified antimicrobial compounds, K_1_ and K_2_. The structures of the compounds, K_1_ and K_2_, were elucidated employing various spectroscopic techniques, including UV, IR, mass and 1D and 2D NMR spectroscopic techniques, resulting in the identification of the compounds, K_1_ and K_2_, as Actinomycin X_2_ and Actinomycin D, respectively. The antimicrobial screening of isolated actinomycins, X_2_ and D, revealed that both actinomycins exhibited potent antimicrobial properties against the tested bacterial strains, and further research revealed that Actinomycin X_2_ exhibited higher antimicrobial property than Actinomycin D. As a result, we concluded that the isolated actinomycete strain, *Streptomyces smyrnaeus* UKAQ_23, is an opulent source for the production of Actinomycin X_2_ and Actinomycin D and could be used as an alternative source of said actinomycins, which could be a significant shift in the pharmaceutical industry^17,47–52^.

Our findings showed that mangrove-derived *Streptomyces smyrnaeus* UKAQ_23 exhibited antimicrobial potential, which is consistent with a previous study in which Tatar et al. reported that *Streptomyces smyrnaeus* sp. nov. isolated from Izmir, Turkey demonstrated antibacterial and antifungal activities against *B.cereus* NRRL B-3711T, *B.pumilus* NRRL-BD 142, *B.subtilis* NRRL B-209, *Candida utilis* NRRL Y-900, and *Aspergillus parasiticus* NRRL-465. However, they were unable to isolate the antimicrobial compounds from that strain^53^.

Our findings of actinomycins, X_2_, and D production from mangrove-derived *Streptomyces smyrnaeus* UKAQ_23 are consistent with previous findings that several *Streptomyces* strains, *e.g., Streptomyces* sp. MS449, *Streptomyces* sp. IMB094, *Streptomyces nasri* YG62, *Streptomyces padanus* JAU4234, *Streptomyces elizabethii.* II, *Streptomyces flavogriseus* NJ-4, *Streptomyces* MITKK-103, *Streptomyces griseoruber, Streptomyces* strain M7, *Streptomyces* sp. HUST012**,** *Streptomyces heliomycini, Streptomyces hydrogenans* IB310 produce Actinomycin X_2_ and Actinomycin D^24,28,47,49–57^.

Our recent findings showed that actinomycins, X_2,_ and D produced by *Streptomyces smyrnaeus* UKAQ_23 exhibited potent antimicrobial activities against MRSA and non-MRSA Gram-positive bacterial strains, but no bioactivity was demonstrated against tested Gram-negative bacteria and fungal strains, which is consistent with previous studies; Sharma and Manhas, who demonstrated the antibacterial potential of actinomycins V, X_2_, and D produced by *Streptomyces* strain M7, and the findings revealed that actinomycins X_2_ and D had significant antibacterial potential against VRE, MRSA, and *Bacillus subtilis* with MIC values ranging from 1.95 to 8.0 μg/ml^54^; Wang et al. demonstrated antimicrobial activity of actinomycins X_0β_, X_2_, and D produced by *Streptomyces heliomycini*, and the results showed that actinomycins X_0β_, X_2,_ and D had substantial antibacterial potential against *S.aureus*, MRSA, *Bacillus subtilis*, and *Bacillus cereus* with MIC values ranging from 0.04 to 2.48 μg/ml^56^.

Wang et al*.* demonstrated the antibacterial potential of four actinomycins, neo-Actinomycin A, neo-Actinomycin B, Actinomycin D, and Actinomycin X_2_ produced by *Streptomyces* sp. IMB094 and the results revealed that actinomycins D and X_2_ had substantial antibacterial activity against *S.aureus*, *S.epidermidis*, and *E.faecalis* with MIC values ranging from 0.06 to 0.5 μg/ml, while neo-Actinomycins A and B exhibited less-substantial antibacterial potential with MIC values ranging, 16–32 μg/ml by neo-Actinomycin A and > 128 μg/ml by neo-Actinomycin B, respectively. However, with MIC values ranging from 16 to 128 μg/ml, all four actinomycins had insignificant antibacterial activity against *E.coli*, *K.pneumoniae*, *P.aeruginosa*, *S.marcescens*, *A.calcoaceticus*, *P.mirabilis*, *P.rettgeri*, *P.vulgaris*, *C.freundii, M.morganii*, *S.maltophilia*, and *Enterobacter* spp.^28^.

Khieu et al*.* demonstrated that Actinomycin D produced by *Streptomyces* sp. HUST012 had substantial antibacterial activity against MRSA ATCC 25923, MRSE ATCC 35984, *Escherichia coli* ATCC 25922, and *Klebsiella pneumoniae* ATCC 13883 with MIC values ranging from 0.04 to 2.24 μg/ml^57^, which was partially deprived of our findings, as our isolated actinomycins had shown non-substantial antibacterial activity against Gram-negative test bacteria. Kulkarni et al*.* demonstrated the antimicrobial potential of Actinomycin D produced by *Streptomyces hydrogenans* IB310. They showed that Actinomycin D had substantial antibacterial and antifungal potential against plant pathogens and suggested the potential application of Actinomycin D in agriculture to manage plants’ bacterial and fungal infections^58^, which was partially deprived of our findings, as our isolated actinomycins had shown non-substantial antifungal activity.

Chen et al*.* reported that several species of *Streptomyces* produce Actinomycin D analogs. However, only a few species produce in significant amount, including *Streptomyces parvulus* (0.152 mg/ml Actinomycin D), *Streptomyces griseoruber* (0.21 mg/ml Actinomycin D), *Streptomyces sindenensis* (0.85 mg/ml Actinomycin D), *Streptomyces* MITKK-103 (0.11 mg/ml Actinomycin X_2_), *Streptomyces* sp. JAU4234 (0.62 mg/ml actinomycin X_2_), *Streptomyces nasri* strain YG62 (0.15 mg/ml Actinomycin X_2_), and *Streptomyces* strain MS449 (1.92 mg/ml Actinomycin X_2_, 1.77 mg/ml Actinomycin D)^24^. This study confirms our findings and suggests that the isolated strain *Streptomyces smyrnaeus* UKAQ 23 is suitable for industrial use as it can produce a significant amount of Actinomycin D. However, prior to its commercial employment, strain improvement is essential.

Our findings on antibiotic production and optimization of fermentation conditions indicated that the modified ISP-4 agar was the best fermentation medium for maximum antibiotic production at pH 6.5, temperature 35 °C, inoculum 5% v/w, agar 1.5% w/v, and incubation period of 7 days. Furthermore, the solid-state fermentation method was superior to the submerged-state fermentation method for the production of crude antibiotics, possibly due to the lack of water in solid-state fermentation. The findings obtained by optimizing the fermentation medium using RSM significantly affected antibiotic production by *Streptomyces smyrnaeus* UKAQ_23. Furthermore, to avoid the formation of fermentation products with poor antibacterial activity, pH, temperature, inoculum concentration, agar concentration, and incubation time, among other variables, must be tightly regulated throughout fermentation. This method may be used to conduct further investigation into antibiotic production. The higher yield of crude antimicrobial extract obtained using the solid-state fermentation method was consistent with previous reports^59–63^. Surprisingly, one study showed that solid-state fermentation conditions are ideal for producing antimicrobial compounds by *Streptomyces youssoufiensis* SF10 strain^64^.

**Figure 10:**
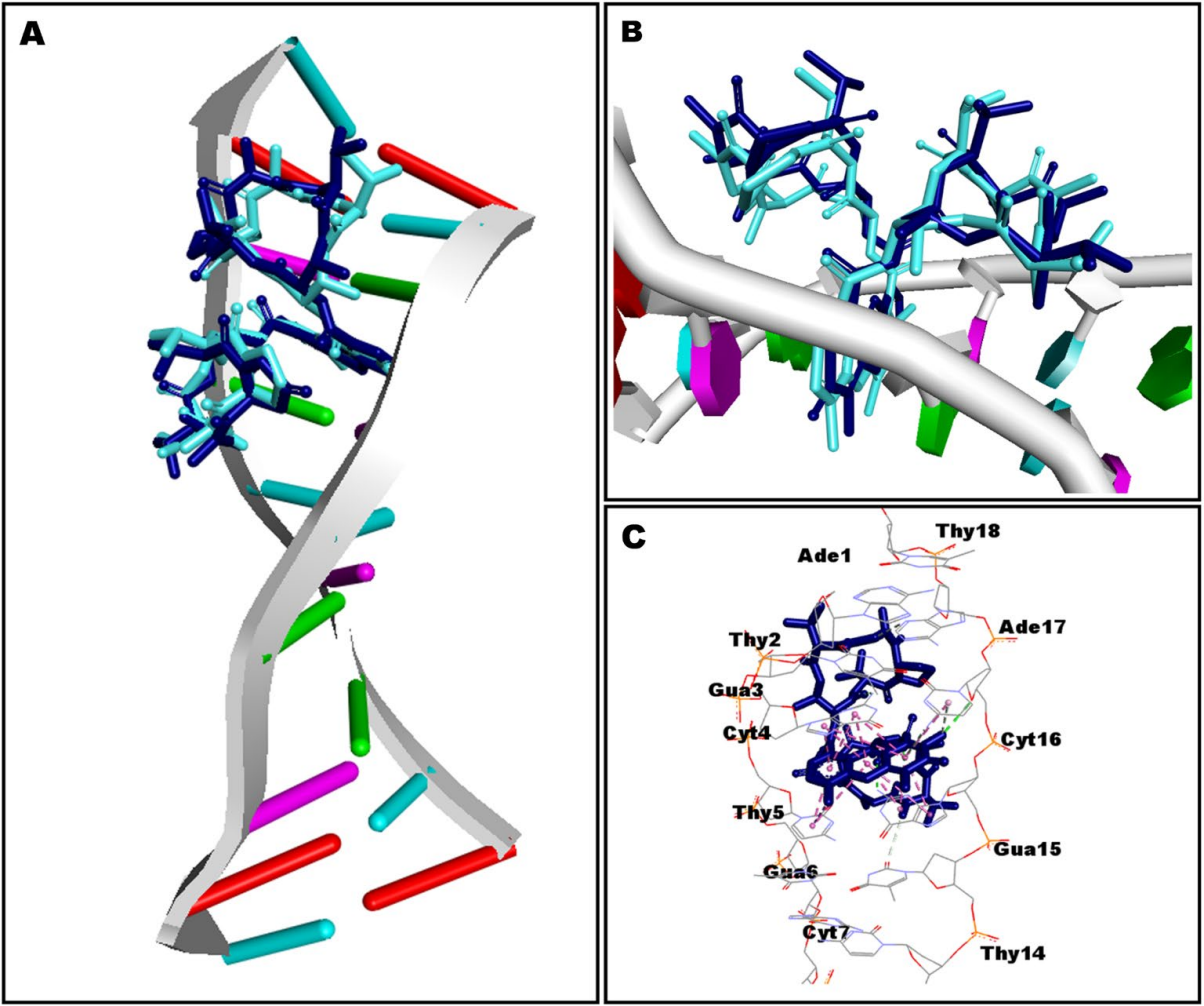
Docked compound K_1_ in the DNA. Co-crystallized actinomycin D is shown in cyan color while docked compound K_1_ is portrayed as a stick in dark blue color. (**A**) Whole DNA fragment used in docking has been shown; (**B**) phenoxazine ring occupying between the base pairs; (**C**) detailed intermolecular contacts are displayed. Biovia Discovery Studio Visualizer 2020 program was used for generating the images (https://discover.3ds.com).

**Figure 11:**
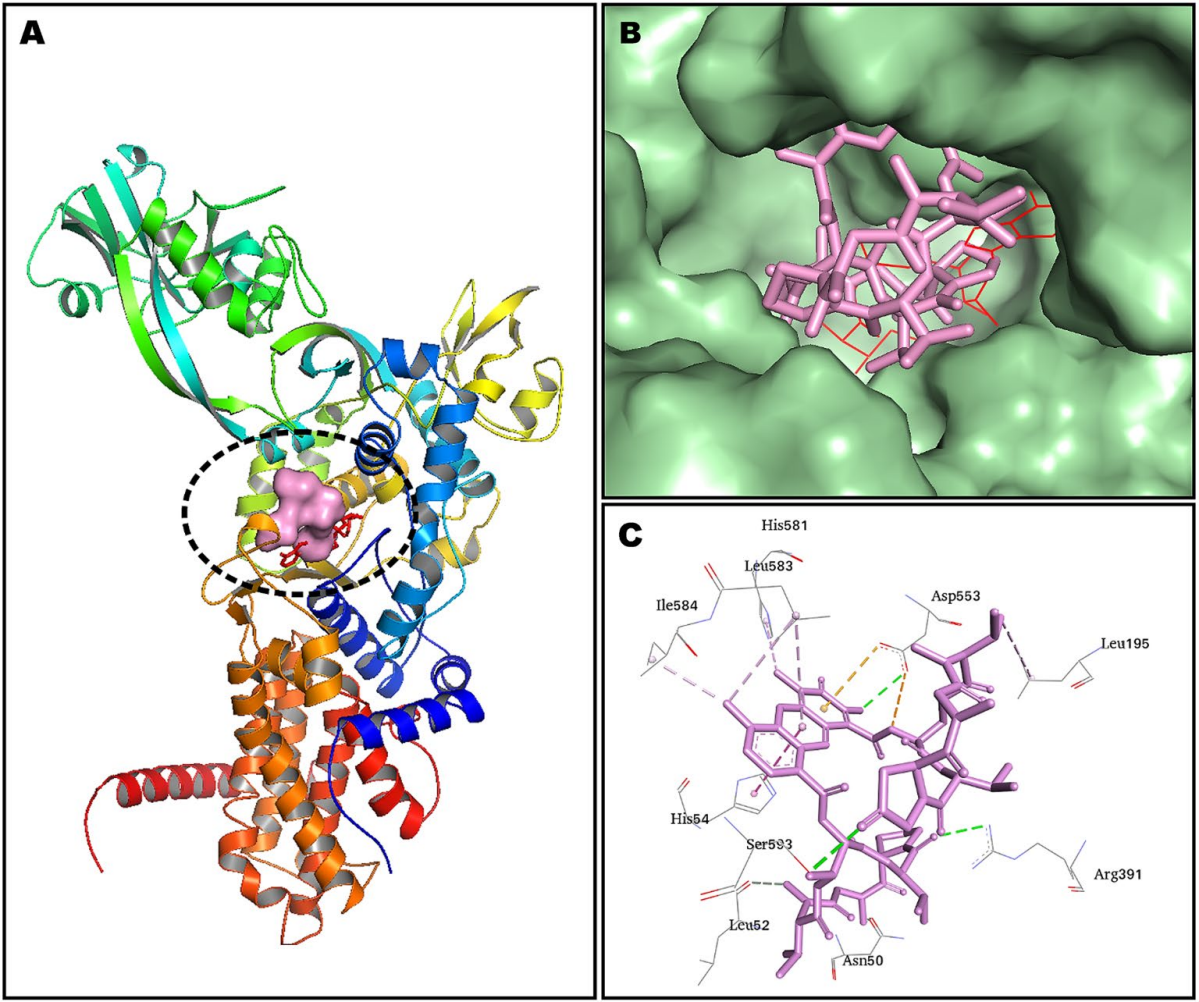
Minimum energy conformation of docked compound K_1_ in the binding pocket of isoleucyl-tRNA synthetase (IleRS). (**A**) IleRS has been shown as cartoon representation while docked compound K_1_ has been shown as a surface in pink color; (**B**) Binding pocket of IleRS has been shown as surface and docked compound K_2_ is represented as a stick in pink while native ligand is demonstrated as line rendering in red color; (**C**) Binding residues are shown inline style and intermolecular interaction are shown as dotted lines. PyMoL 2.4.1 (https://pymol.org/2/) (**A**,**B**) and Biovia Discovery Studio Visualizer 2020 (C) programs were used for generating the images.

Molecular docking is a robust computational technique for predicting bound conformations of drug candidates and their binding affinities. In this study, the molecular interaction of compounds K_1_ and K_2_ against several drug-targets of bacterial biochemical pathways was studied for the first time by molecular docking using AutoDock Vina 1.1.2^65,66^. Analysis of the docking results (Table 6) revealed that both compounds, K_1_ and K_2,_ possessed sufficient molecular framework for their interaction with numerous drug targets, which might be considered accountable for producing the antibacterial activity (Figs. 10–11). The preferential binding of compound K_1_ with IleRS, MurD ligase, and GFAT targets further signifies the importance of an additional keto group in the pyrrolidine ring of the proline moiety. Although both compounds K_1_ and K_2_ were observed to be most potent against IleRS, it was compound K_1_ which offered an additional hydrogen bond accepting platform in the form of oxo-Pro moiety for interaction with Ser593 residue in the binding pocket of IleRS, the most promising antibacterial drug target as per our docking results (Fig. 11). However, PBP-1a was the least desirable macromolecular site for both compounds K_1_ and K_2_.

Among potential contacts of compound K_2_ within inhibitor binding cavity of IleRS, Asn50, Leu52, Arg391, Gly551, His581, and Ser593 were recognized to afford polar interactions in the form of hydrogen bonds. Residue His581 is positioned at the C-terminus of the last β-sheet of the Rossmann fold of the IleRS protein, is conserved in the bacterial IleRS but is substituted with an asparagine/serine in the eukaryotic IleRS^36^. Both compounds K_1_ and K_2_ interacted with His581 by polar and hydrophobic contacts, respectively. IleRS plays an important role in the tRNA aminoacylation step of the bacterial protein synthesis, and hence, its inhibition has proven to be an effective antimicrobial strategy, hampering a vital step of protein synthesis^67^. These results may provide a foundation for experimental design for further exploration of the precise antimicrobial mechanism of action of compounds K_1_ and K_2_.

## Methods

### Sample collection and isolation of actinomycetes

A total of twenty-five mangrove sediment samples were collected from Jubail, Saudi Arabia (latitude: 27°00′40″ N; longitude: 49°39′29″ E; altitude: 22 ft; annual rainfall: 97 mm; average temperature: 26.6 °C)^8^. All the mangrove sediment samples were air-dried at room temperature for 1 week and then pre-treated for 1 h in a hot air oven at 60 °C to enrich and selectively isolate actinomycetes^68^. A serial dilution of up to 10^−5^ was then prepared by dissolving the 1 g of sample in 0.89% NaCl. From each dilution, a 0.1 ml sample was spread out on ISP-4 agar supplemented with nalidixic acid (50 μg/ml) and cycloheximide (50 μg/ml) to isolate actinomycetes strains. The nalidixic acid and cycloheximide were supplemented to inhibit the growth of the Gram-negative bacteria and fungi, respectively^8,68^. The inoculated plates were then incubated at 28 °C for up to 14 days. The isolated actinomycetes were sub-cultured and purified on ISP-4 slants. The purified cultures were preserved in 20% (v/v) glycerol at − 78 °C for further use.

### Preliminary antimicrobial screening of isolated actinomycetes

Preliminary antimicrobial screening of the isolated actinomycetes was conducted by the spot inoculation method using modified tryptic soy agar (MTSA) plates^3,4,8^. MTSA plates consisted of starch soluble 1 g, the pancreatic digest of casein 1.5 g, papain digest of soya bean 0.5 g, sodium chloride 0.5 g, agar 1.7 g, peptone 0.4 g, tryptone 0.4 g, beef extract 0.3 g, lactose 1 g, L-cystine 0.013 g, bromothymol blue 0.002 g, agar 1.5–1.7 g, ultrapure water 100 ml, and pH 6.8 ± 0.2.

For preliminary antimicrobial screening, 10 Gram-positive bacteria, *Staphylococcus aureus* (*S.aureus*) ATCC 29213, MRSA-A, MRSA-B, MRSA-C, *Staphylococcus saprophyticus* (*S.saprophyticus)* ATCC 43867, *Staphylococcus epidermidis* (*S.epidermidis*) ATCC 12228, *Streptococcus pyogenes* (*S.pyogenes)*-A ATCC 19615, *Streptococcus pneumoniae* (*S.pneumoniae*) ATCC 49619, *Enterococcus faecalis* (*E.faecalis*) ATCC 29212, *Bacillus cereus* (*B.cereus*) ATCC 10876), 7 Gram-negative bacteria, *Escherichia coli* (*E.coli*) ATCC 25922, *Klebsiella pneumoniae* (*K.pneumoniae*) ATCC 27736, *Pseudomonas aerugenosa* (*P.aerugenosa*) ATCC 9027, *Proteus mirabilis* (*P.mirabilis*) ATCC 29906, *Proteus vulgaris* (*P.vulgaris*) ATCC 6380, *Salmonella typhimurium* (*S.typhimurium*) ATCC 13311, *Shigella flexneri* (*S.flexneri*) ATCC 12022), and 2 fungal species *Candida albicans* (*C.albicans*) ATCC 10231 and *Aspergillus niger* (*A.niger*) ATCC 6275 were used as test organisms. The antibacterial activity was determined by incubating the inoculated plates at 35 ± 2 °C for 24 h., and antifungal activity was determined by incubating the inoculated plates at 28 ± 2 °C for up to 48 h. The antimicrobial activities of all the isolates were recorded by observing the zone of inhibition around the actinomycetes spot^8,68^. The selection of the highly potent actinomycete strain, UKAQ_23, was based on the zone of inhibition around the inoculated spot (growth).

### Identification and characterization of selected isolate UKAQ_23

The selected isolate UKAQ_23 was identified by 16S rRNA gene sequencing^8,24^. The phylogenetic analysis was performed using the Maximum Likelihood approach using the Tamura-Nei model, and the phylogenetic tree was constructed using the neighbor-joining (NJ) method MEGA X^24,69^.

The cultural characterization of strain UKAQ_23 was determined by recording the cultural characteristics, *i.e.*, the color of substrate and aerial mycelium, spore formation, diffusible pigment on various growth media, *i.e.*, GM-1… GM-7) at 28 °C for up to 7 days. SEM recorded the morphological characteristics^54^.

The physiological characterization of strain UKAQ_23 was performed by determining the effects of various physicochemical factors, *e.g*., assimilation of different carbon sources (dextrose, galactose, maltose, lactose, sucrose, dextrin, starch soluble, starch maize, mannitol, sorbitol, glycerol), nitrogen sources (L-asparagine monohydrate, L-glutamine, glycine, L-leucine, L-methionine, L-tryptophan, ammonium sulfate, ammonium chloride, ammonium oxalate, potassium nitrate, cornmeal, soya meal), tolerance of NaCl concentrations (0.25–10% w/v with an increment of 0.25%), pH (5.5–10 with an increment of 0.5), temperature (25–55 °C with an increment of 5 °C), and incubation periods (1–10 days with an increment of 24 h.) on the growth of UKAQ_23^70–75^. The biochemical characterization was performed to determine UKAQ_23's ability to produce various hydrolytic enzymes. The antibiotic susceptibility pattern was determined by the disc diffusion method with amoxycillin (10 µg), ceftriaxone (30 µg), chloramphenicol (30 g), clindamycin (2 µg), imipenem (10 µg), and tetracycline (30 µg)^54,76–78^^.^

### Antibiotic production

Initially, antibiotic production was carried out in four different fermentation media, *i.e.*, ISP-1 broth, ISP-2 broth, ISP-4 broth, and 1% (w/v) starch tryptic soy broth. The best fermentation medium (ISP-4) was selected based on its antimicrobial activity. The well diffusion method determined the antimicrobial activity of fermented broth^4^. The effects of different carbon and nitrogen sources on antibiotic production were determined in ISP-4 base fermentation media, *i.e.*, FM-1… FM-24 (see, Supplementary Table S9), and during this fermentation, the basal fermentation conditions were kept constant, *i.e.*, pH 6.5, temperature 30 °C, inoculum 5% v/w, shaking speed 250 rpm, and incubation period 7 days. Fermentation was carried out in submerged-state fermentation^2,24,53^. The best fermentation media (FM) were selected for further finer optimization based on their antimicrobial activity demonstrated against the test organism. The antimicrobial activity of each fermented broth was determined by the well-diffusion method using 80 μl of cell-free broth/well against the test organism (*S. aureus* ATCC 29213). The zone of inhibition was measured on an mm scale^4^.

### Optimization of media composition and fermentation conditions for high yield antibiotic production

Initially, the production of antibiotic was carried out in submerged-state fermentation, but owing to large fluctuation in the production of antibiotic, the optimization was shifted to solid-state fermentation^61,62^. The effects of various carbon source concentrations (0.2–1% w/v with an increment of 0.2%), nitrogen source concentrations (0.25–1% w/v with an increment of 0.25%), NaCl concentrations (0.5–2% w/v with an increment of 0.25%), K_2_HPO_4_ concentrations (0.3–1.5% w/v with an increment of 0.3%), CaCl_2_ concentrations (0.05- 0.40% w/v with an increment of 0.05%), MgSO_4_ concentrations (0.05–0.40% w/v with an increment of 0.05%), CaCO_3_ concentrations (0.005–0.030% w/v with an increment of 0.005%), FeSO_4_ concentrations (0.005–0.030% w/v with an increment of 0.005%), ZnSO_4_ concentrations (0.005- 0.030% w/v with an increment of 0.005%), pH (6–7.5 with an increment of 0.5), temperature (25–40 °C with an increment of 5 °C), inoculum concentrations (2.5–10%, with an increment of 2.5%), agar concentrations (1–2% w/v with an increment of 0.25%), and incubation periods (1–10 days with an increment of 24 h.) were determined.

The effects of media constituents and fermentation conditions on antibiotic production were determined by varying the one factor at a time and base fermentation conditions were kept constant, *i.e*., pH 6.5, temperature 30 °C, carbon source 1% w/v, nitrogen source 0.5% w/v, NaCl 0.50% w/v, inoculum 5% v/w, agar 1.5% w/v, and incubation period of 7 days. After completing each set of fermentation, the yield of antimicrobial extract was measured in mg/kg of fermented agar^53,61,62,73,74^.

### Optimization of antibiotic production by RSM

After optimizing the media composition and fermentation conditions, the four most influential variables (pH, temperature, inoculum volumes, and agar concentrations) were chosen for further antibiotic-production-optimization by RSM using the Box-Behnken design (BBD)^79^. The predicted model was validated and found to be statistically suitable for use. A total of 27 experiments were carried out to optimize the main factors for a 3-levels-4-factors BBD with three replicates at the center. After evaluating the responses for each trial, each response was fitted to an individual second-order polynomial model. For media optimization, a total of twenty-seven BBD experiments were run in one block. The minimum and maximum ranges of variables investigated in terms of their actual and coded values are mentioned (see, Supplementary Table S10). To assess the impact of process variables on antibiotic production, the BBD was used (see, Supplementary Table S11).

### Extraction and purification of antimicrobial compounds

The extraction of antimicrobial compounds from the fermented agar was carried out by solid–liquid extraction method as described earlier^60,61^ (see, Supplementary Figure S22). The resulted crude antimicrobial extract was subjected to further purification.

A thin layer chromatography (TLC) was employed to assess the purity and number of compounds present in the crude antimicrobial extract using a solvent system composed of dichloromethane (CH_2_Cl_2_) and acetone ((CH_3_)_2_CO) in a ratio of 60:40. The TLC was run on pre-coated silica gel (60 F254, Merck, USA) sheets^2^. The plates were visualized under UV light. The bioautography was performed on a developed TLC plate^80^. *S.aureus* ATCC 29213 was employed as a test organism.

The purification of antimicrobial compounds was carried out by employing the preparative TLC plates (60 F254, 1000 µm, 20 × 20 cm, Miles Scientific, USA), size-exclusion chromatography (Sephadex LH-20; GE Healthcare Bio-Sciences AB, Uppsala, Sweden), and silica gel (60 Å, 0.071–0.16 mm, MACHEREY–NAGEL, Germany) column chromatography^54,81,82^.

A solvent system composed of dichloromethane and acetone (60:40) was employed in the preparative TLC and silica gel column chromatography, while pure methanol was employed in the size-exclusion chromatography. The purification steps culminated into two pure antimicrobial compounds named K_1_ and K_2_. The homogeneity of purified compounds was assessed by HPLC (Ultimate 3000 HPLC, Thermo Scientific, Waltham, Massachusetts, USA).

### Physicochemical characterization and structure elucidation of purified antimicrobial compounds K_1_ and K_2_

The physicochemical characterization and structure elucidation of isolated antimicrobial compounds, K_1_ and K_2,_ were carried out by recording the color, appearance, solubility, melting point (°C), UV–Visible (λ_max_, nm) absorbance, FT-IR (υ_max_, cm^−1^) absorbance, monoisotopic masses, ( +)-HR-ESI-MS, LC–MS, LC–MS-MS, 1D (^1^H, ^13^C, DEPT), and 2D (COSY, HSQC, HMBC) NMR spectroscopy^17,23,25–28,83–85^.

The solubility of compounds was determined by dissolving the compounds in various solvents, *e.g.*, water, ethanol, methanol, acetone, acetonitrile, dimethylformamide, dimethyl sulphoxide, chloroform, ethyl acetate, dichloromethane, and n-hexane at a concentration of 10 mg/ml. The melting points of the compounds were determined by the capillary method with the Dynalon DMP100 Digital Melting Point Device (Spectrum Chemical Manufacturing Corporation, USA). The compounds were dissolved in methanol at a concentration of 1 mg/ml, and UV–Visible spectra were recorded with the Ultraspec 8000 spectrophotometer (GE, Pittsburgh, PA, USA). FT-IR spectra were recorded with the Nicolet iS20 FT-IR spectrometer (Thermo Scientific, Waltham, Massachusetts, USA). The MS^n^ analysis was performed on LCMS-IT-TOF (Shimadzu Corporation, Japan). The instruments parameters were as follows: Positive-ion mode, calibration with the Tune Solution, mass accuracy was better than 5 ppm, scan range: 50–2000 *m/z,* drying gas: nitrogen (flow rate: 1.5 l/min), temperature: 200 °C, solvents: isocratic elution 50% B in A (A = 0.1% HCOOH in water; B = 0.1% HCOOH in MeCN), flow rate: 0.1 µl/min, injection volume: 1 µl. LC-MS analysis was performed on Shimadzu LCMS-9030 quadrupole time-of-flight (Q-TOF). Separation was carried out on an Aeris Peptide XB-C18 (100 mm x 2.1 mm, 3.6 µm) column. Eluent A: 0.1% formic acid in H_2_O, eluent B: 0.1% formic acid in MeCN. The gradient conditions (B%) were from 5 to 85% B in 15 min. Flow rate: 0.1 ml/min, injection volume: 1 µl. The NMR spectra were recorded on the Bruker 700 MHz AVANACIII NMR spectrometer equipped with Bruker CPTCI multinuclear probe. Topspin 4.0.4 software (Bruker BioSpin, Rheinstetten, Germany) was used for both the data collection and spectral processing. The obtained data were compared with the standard actinomycins, X_2_ and D, and previously reported NMR and MS data of the actinomycins.

### Antimicrobial activity

#### Primary antimicrobial activity

The standard disc diffusion method determined the primary antimicrobial activity of the isolated antimicrobial compounds (K_1_, K_2_, and K_1_ + K_2_)^76–78^. The isolated antimicrobial compounds were dissolved in methanol, and then diluted samples were dispensed on sterile paper discs (6 mm size). Each disk consisted of 5 µg of the antimicrobial compound. This experiment investigated the antimicrobial efficacy of isolated antimicrobial compounds on the tested microorganisms. A disk containing 20 μl of methanol was used as a negative control, while Levofloxacin (5 µg/disc) was used as positive control antibiotic. Each test was performed in triplicate. The diameters of the inhibitory zones were measured on an mm scale. The results were recorded in Mean ± Standard Deviation (SD).

#### Secondary antimicrobial activity (MIC and MBC)

The secondary antimicrobial activity of isolated antimicrobial compounds (K_1_ and K_2_) was determined by performing the MIC and MBC. MIC was determined by the resazurin-based micro broth dilution method, while MBC was determined by standard spot inoculation method^3,4,78,86^. The antimicrobial compounds were dissolved in methanol at a concentration of 200 µg/ml, and then, various concentrations (0.098–50 µg/ml) were prepared in Mueller–Hinton broth (MHB) by following the two-fold serial dilution method in microtiter plates (columns 2–11). Levofloxacin (5 µg/ml) was used as a control antibiotic (column 1). A 100 µl suspension of each test organism (0.5 McFarland) was dispensed in its respective well in columns 1-11. Each well in columns 1-11 contained an equal volume of the test antibiotics and suspensions of the test bacteria. Following the addition of the bacterial suspensions, the plates were incubated at 35 °C for 18-24 h. After incubation, 30 μl of sterile resazurin dye (0.015% w/v) was dispensed into each well of columns 1-11. The plates were kept at room temperature for 5 h. After 5 h, the results of MIC were recorded. The prepared concentrations of the antimicrobial compounds were evaluated for their antimicrobial efficacy against selected test organisms. The lowest concentration of the tested antimicrobial compound showed no color change from blue to pink was considered MIC. The lowest concentration of the tested antimicrobial compound showed no isolated colony on the inoculated plate considered MBC. The results were expressed in µg/ml.

### Statistical analyses

The statistical software package Minitab 19.2020.1 was used to analyze the experimental design applied in RSM during optimization of fermentation conditions for maximum yield of antibiotic. A One-Way ANOVA statistical test statistically analyzed the results of primary antimicrobial activity of the isolated antimicrobial compounds K_1_ and K_2_ to determine the statistical differences among the means of groups (tested bacteria and isolated actinomycins). The post hoc test (Tukey method) was performed to determine the significance of interactions among the means of groups, where p = 0.05 was considered as statistically significant. The statistical analyses were performed with SPSS software, version 20.0 (IBM, USA)^52,87^.

### Molecular docking studies

X-ray crystal structures of several protein targets known to be associated with antimicrobial biological activity elicitation and employed in antimicrobial drug discovery and development, e.g., DNA gyrase, dihydropteroate synthase, dihydrofolate reductase, glucosamine-fructose-6-phosphate aminotransferase, isoleucyl-tRNA synthetase, tyrosyl-tRNA synthetase, penicillin-binding protein-1a, and UDP-N-acetylmuramoyl-L-alanine: D-glutamate ligase were retrieved from the Research Collaboratory for Structural Bioinformatics Protein Data Bank (RCSB PDB, http://www.rcsb.org/pdb/home/home.do) with PDB IDs 3TTZ, 2VEG, 3SRW, 2VF5, 1JZQ, 1JIJ, 3UDI and 2X5O. Also, Actinomycin D co-crystallized with DNA fragment (PDB ID: 1MNV) was also included in the study. Biovia Discovery Studio Visualizer 2020 and MGLTools 1.5.6 were used for the preparation of receptors^88^.

All the co-crystalized ligands, water molecules, and cofactors were deleted, and Gasteiger charges were added to each receptor individually. Two-dimensional chemical structures of the compounds K_1_ and K_2_ were drawn in ChemDraw Ultra and converted to their three-dimensional coordinate by using the Chem3D Ultra program, energy minimized by MM2 method saved in PDB format. All non-polar hydrogens of the ligands were merged, and rotatable bonds were defined in MGL Tools 1.5.6. AutoDock Vina 1.1.2 was used for molecular docking simulation using default protocol with exhaustiveness adjusted to 12^65^.  In each receptor, a grid box having dimensions of 30 points in all directions was built with a grid spacing of 1 Å at the center of respective co-crystallized ligands. At the end of the docking computation, the best poses were selected from the top ten models from each target by examining their binding energy (ΔG binding, kcal/mol) and non-bond interactions profile^89,90^. Molecular interactions analyses were performed in Biovia Discovery Studio Visualizer 2020^91^, and PyMoL 1.7.4^92^ programs.

### Patent

A patent has been filed on January 22, 2021, at the office of Intellectual Property of India. The patent filing reference number is 202111003185. The final decision is awaited.

## Supplementary Information

Supplementary Information.
