# Supplementary material for: Isolation, characterization, anti-MRSA evaluation, and in-silico multi-target anti-microbial validations of actinomycin X2 and actinomycin D produced by novel Streptomyces smyrnaeus UKAQ_23
Source: Sci Rep. 2021 Jul 15;11:14539. doi: 10.1038/s41598-021-93285-7 (PMC8282855; doi:10.1038/s41598-021-93285-7)
Supplement: Supplementary file 1 — Supplementary Information. [file 41598_2021_93285_MOESM1_ESM.doc]

# Supplementary dataset files

# Isolation, Characterization, Anti-MRSA Evaluation, and *In-Silico* Multi-Target Anti-Microbial Validations of Actinomycin X_2_ and Actinomycin D Produced by Novel *Streptomyces smyrnaeus* UKAQ_23

# Kamal A. Qureshi^1,2*^, Avinash D. Bholay^3^, Pankaj K. Rai^1^, Hamdoon A. Mohammed^4,9^, Riaz A. Khan^4^, Faizul Azam^5^, Mariusz Jaremko^6*^, Abdul-Hamid Emwas^7^, Piotr Stefanowicz^8^, Mateusz Waliczek^8^, Monika Kijewska^8^, Ehab A. Ragab^9^, Medhat Rehan^10,11^, Gamal O. Elhassan^2^, Mohammed J. Anwar^12^, and Dinesh K. Prajapati^1*^

^1^Faculty of Biosciences and Biotechnology, Invertis University, Bareilly, UP, 243123, India

^2^Department of Pharmaceutics, Unaizah College of Pharmacy, Qassim University, Unaizah, Qassim, 51911,

Kingdom of Saudi Arabia

^3^Department of Microbiology, KTHM College, Savitribai Phule Pune University, Nashik, MS, 42202, India

^4^Department of Medicinal Chemistry and Pharmacognosy, College of Pharmacy, Qassim University, Buraydah,

Qassim, 51452, Kingdom of Saudi Arabia

^5^Department of Pharmaceutical Chemistry and Pharmacognosy, Unaizah College of Pharmacy, Qassim University, Unaizah, Qassim, 51911, Kingdom of Saudi Arabia

^6^Biological and Environmental Sciences and Engineering Division (BESE) Division, King Abdullah University of

Science and Technology (KAUST), Thuwal, 23955-6900, Kingdom of Saudi Arabia

^7^Core Labs, King Abdullah University of Science and Technology (KAUST), Thuwal, 23955-6900, Kingdom of

Saudi Arabia

^8^Faculty of Chemistry, University of Wroclaw, F. Joliot-Curie, Street-14, Wroclaw, 50-383, Poland

^9^Department of Pharmacognosy, Faculty of Pharmacy, Al-Azhar University, Cairo, 11371, Egypt

^10^Department of Genetics, Faculty of Agriculture, Kafr El-Sheikh University, Kafr El-Sheikh, 33516, Egypt

^11^Department of Plant Production and Protection, College of Agriculture and Veterinary Medicine, Qassim

University, Buraydah, Qassim, 51452, Kingdom of Saudi Arabia

^12^Department of Pharmacology and Toxicology, Unaizah College of Pharmacy, Qassim University, Unaizah,

Qassim, 51911, Kingdom of Saudi Arabia

**Correspondences: ka.afrah@gmail.com (K.A.Q.); dinesh.p@invertis.org (D.K.P.); mariusz.jaremko@kaust.edu.sa (M.J.)*


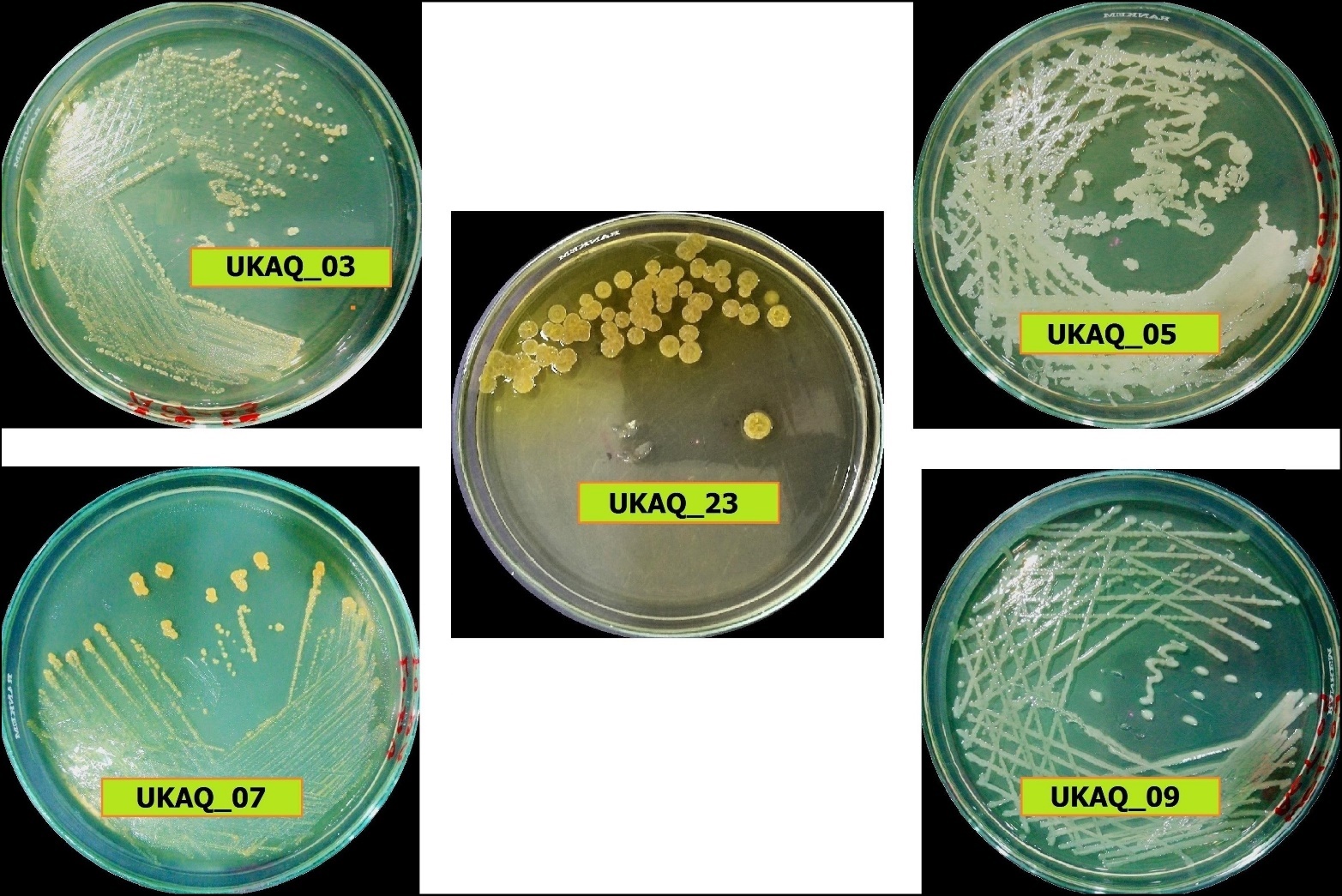


**Figure S1.** Growths of isolated actinomycetes strains on ISP-4 agar at 28 °C for 7 days


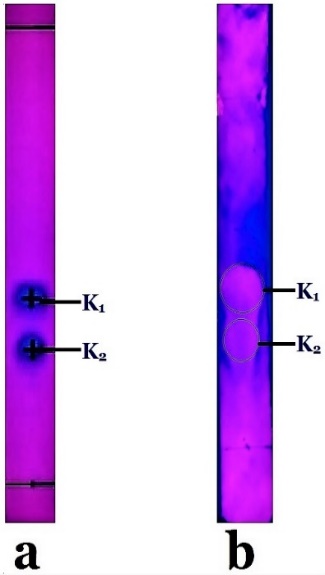


Figure S2. (a) two prominent compounds, K_1_ and K_2_ on TLC sheet; (b) bioautography sheet exhibiting inhibitory zones around the separated compounds


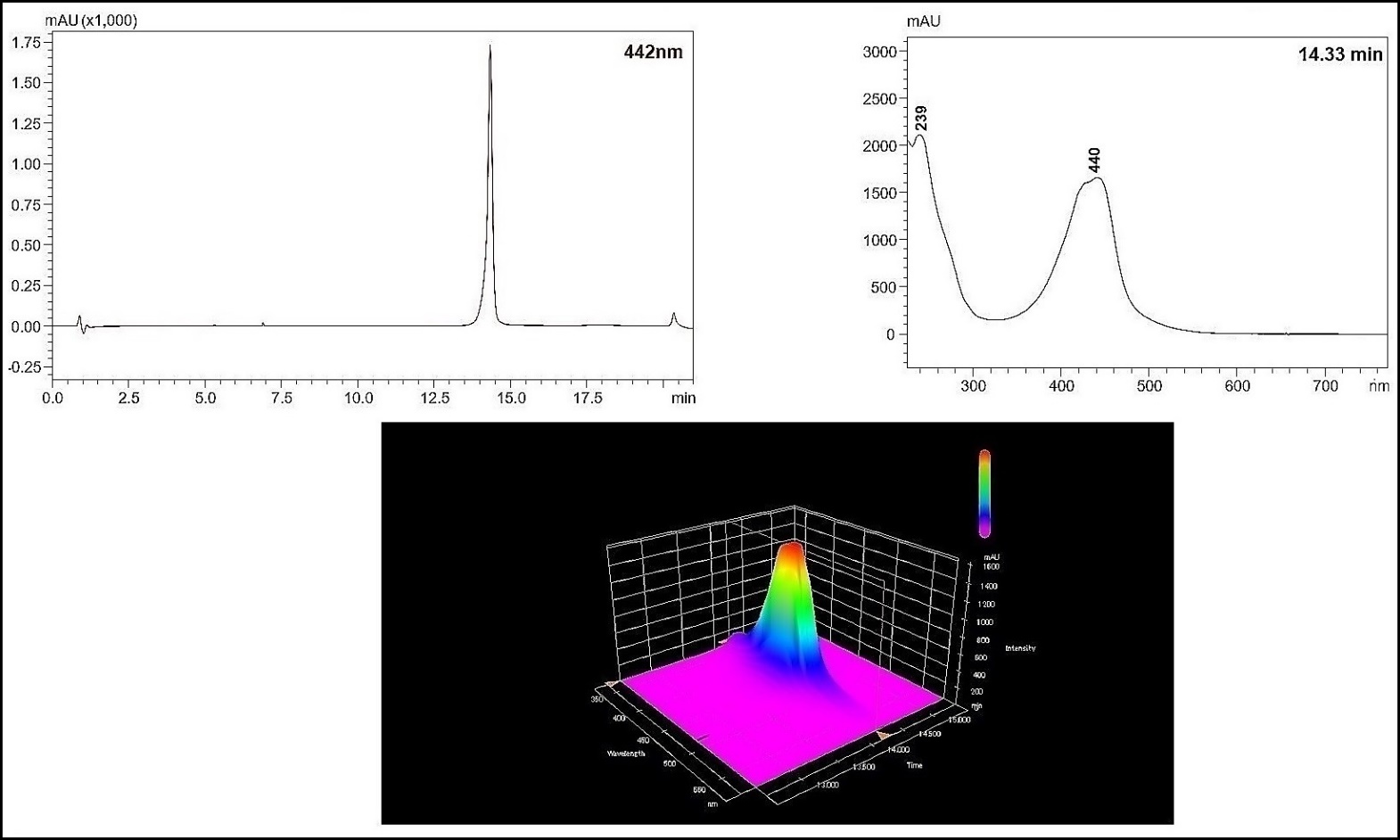


**Figure S3.** UV-Visible spectrum of compound K_1_.


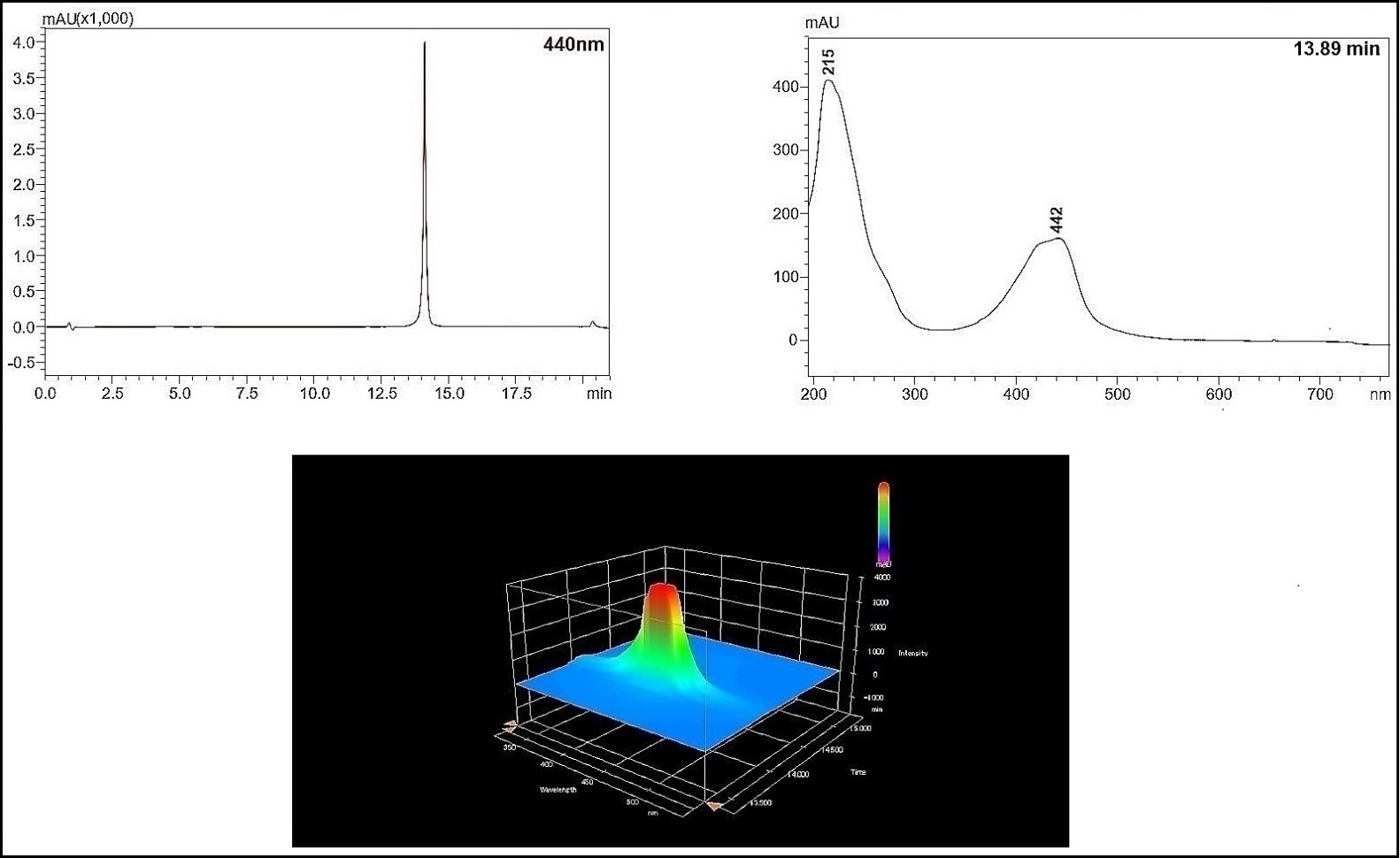


**Figure S4.** UV-Visible spectrum of compound K_2_.


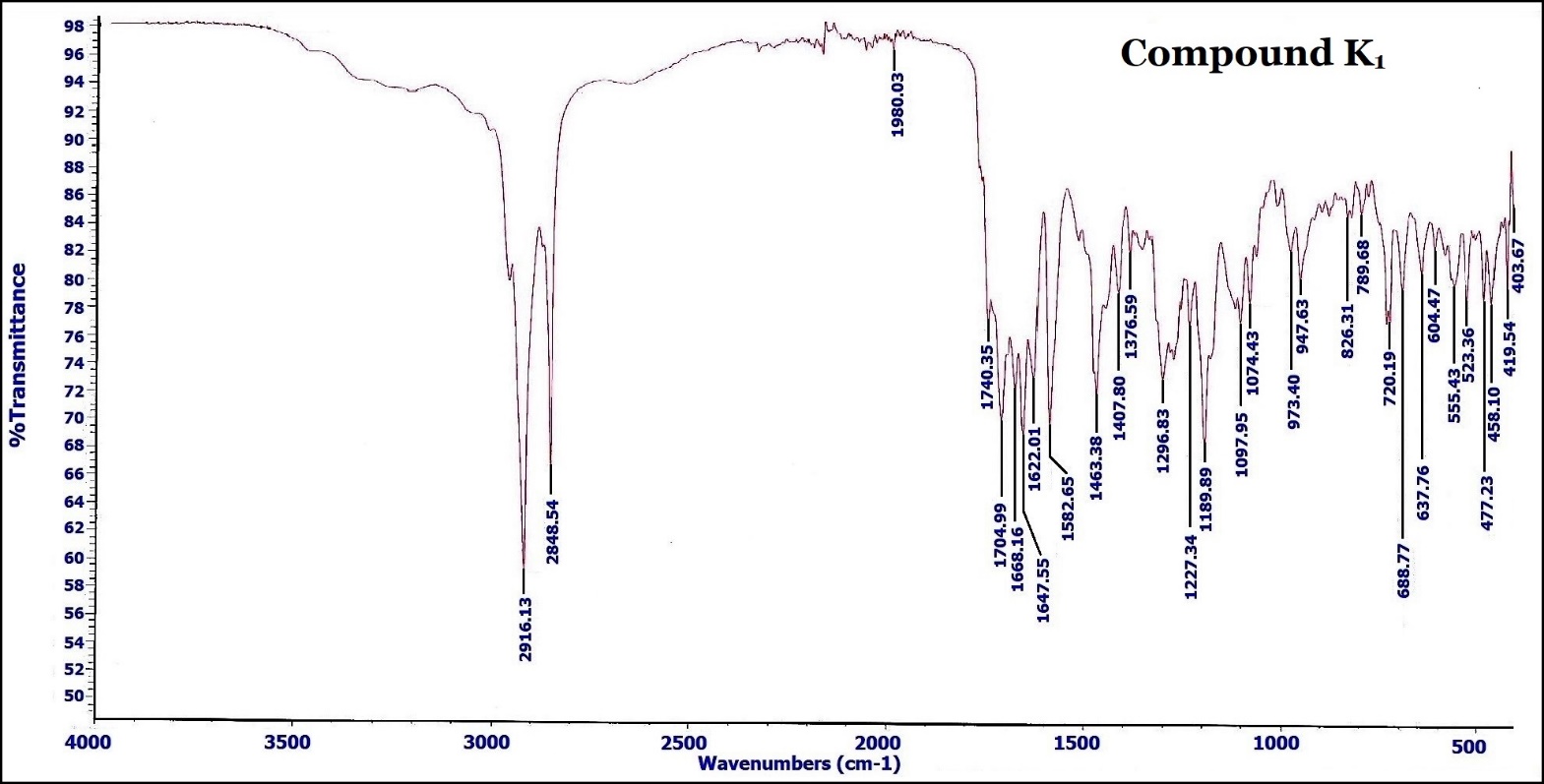


**Figure S5.** FTIR spectrum of compound K_1_.


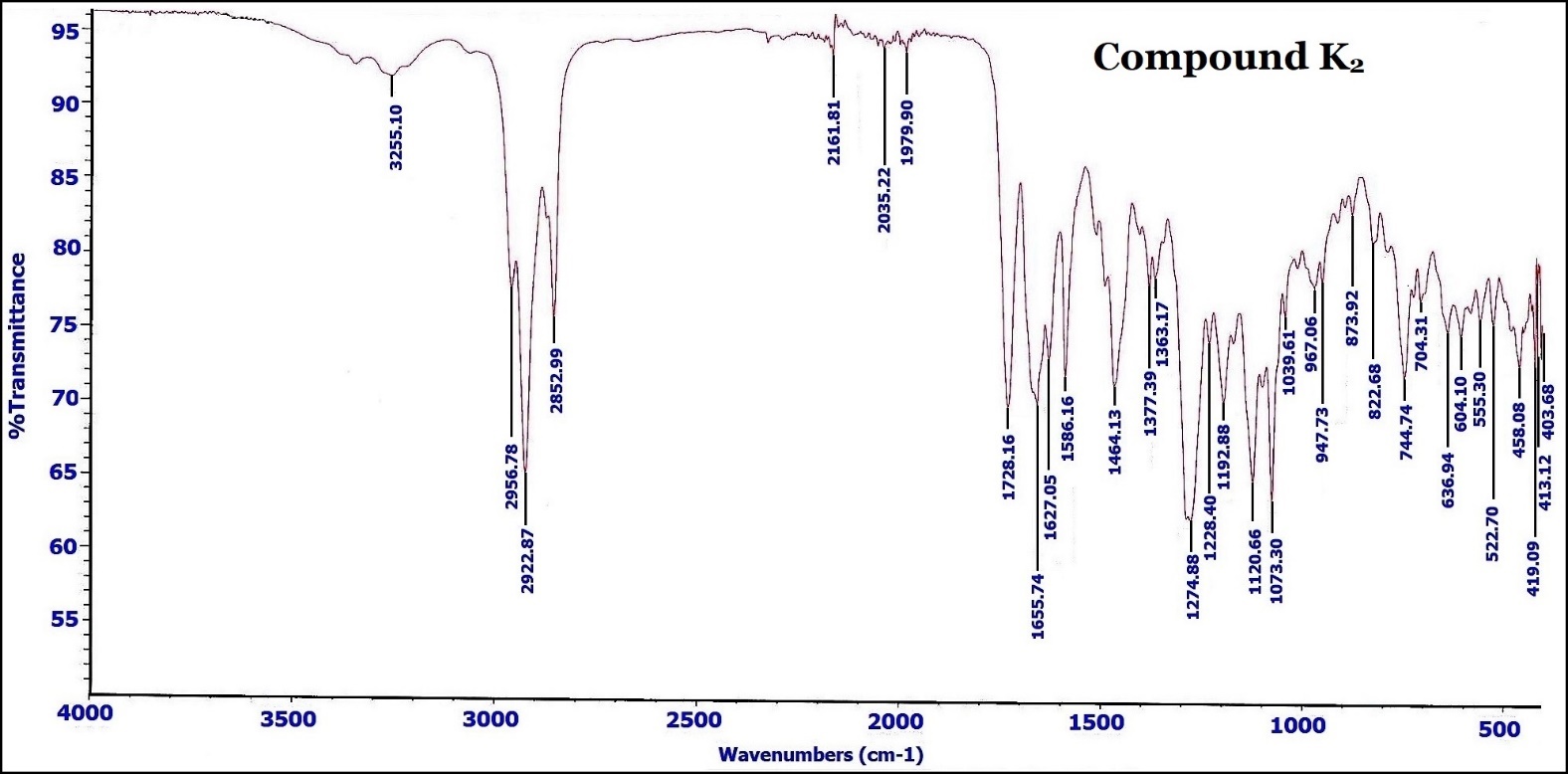


**Figure S6.** FTIR spectrum of compound K_2_.


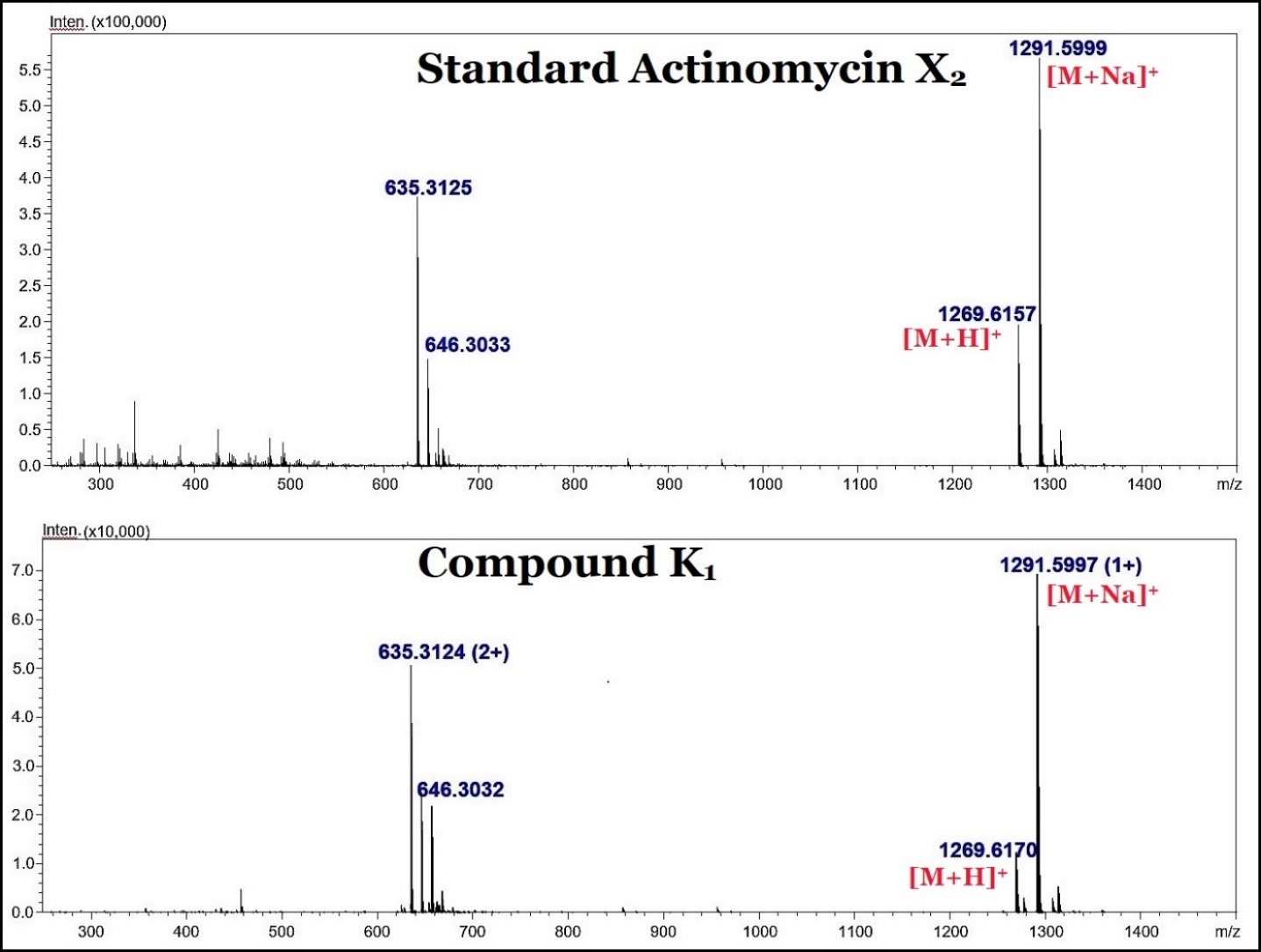


**Figure S7.** Comparison of HRESIMS spectra of compound K_1_ with standard Actinomycin X_2_.

**
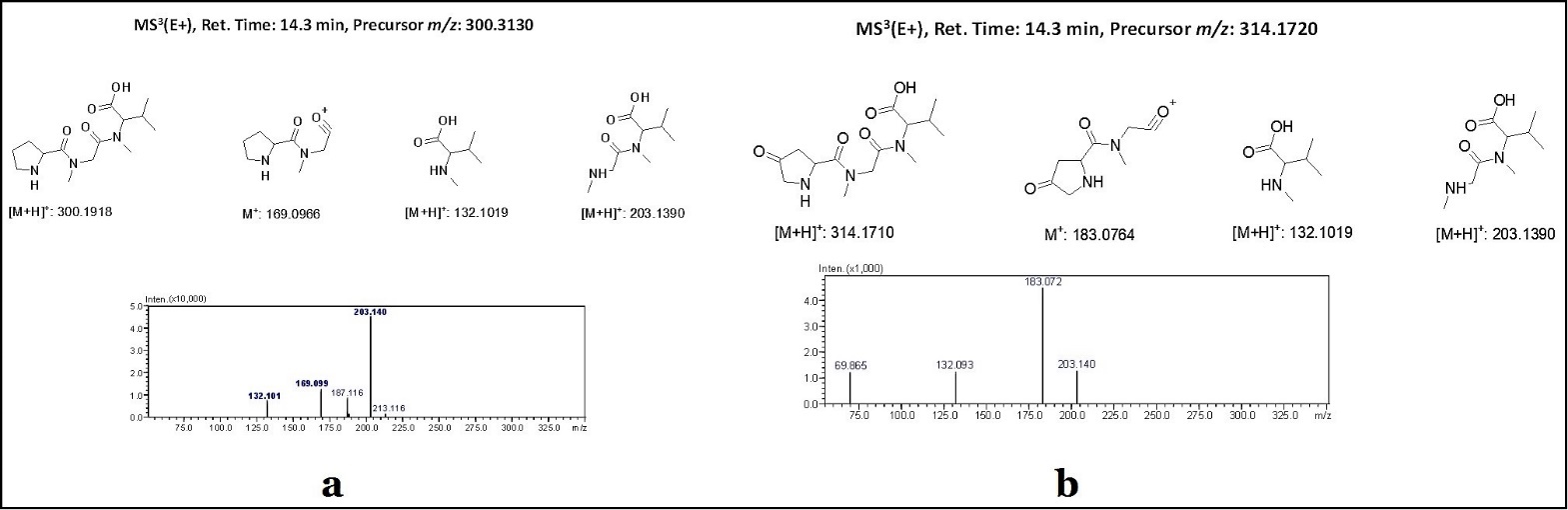
**

**Figure S8.**  MS^3^ analyses of 300.313 and 314.172 ions generated by MS^2^ of ion at m/z 635.3125 [M + 2H]^2+^.


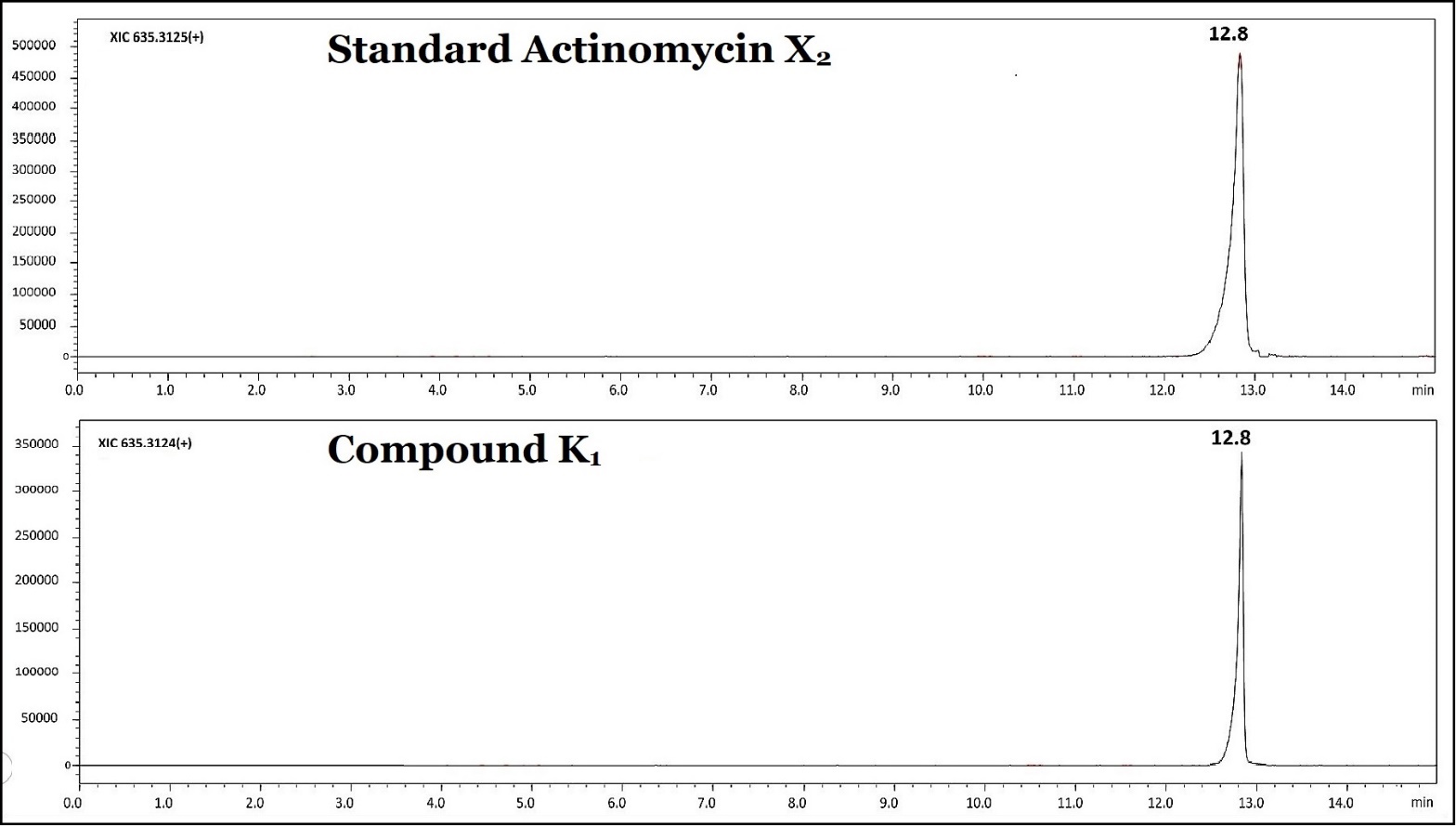


**Figure S9.** LC-MS comparison (retention time) of compound K_1_ with standard Actinomycin X_2_.


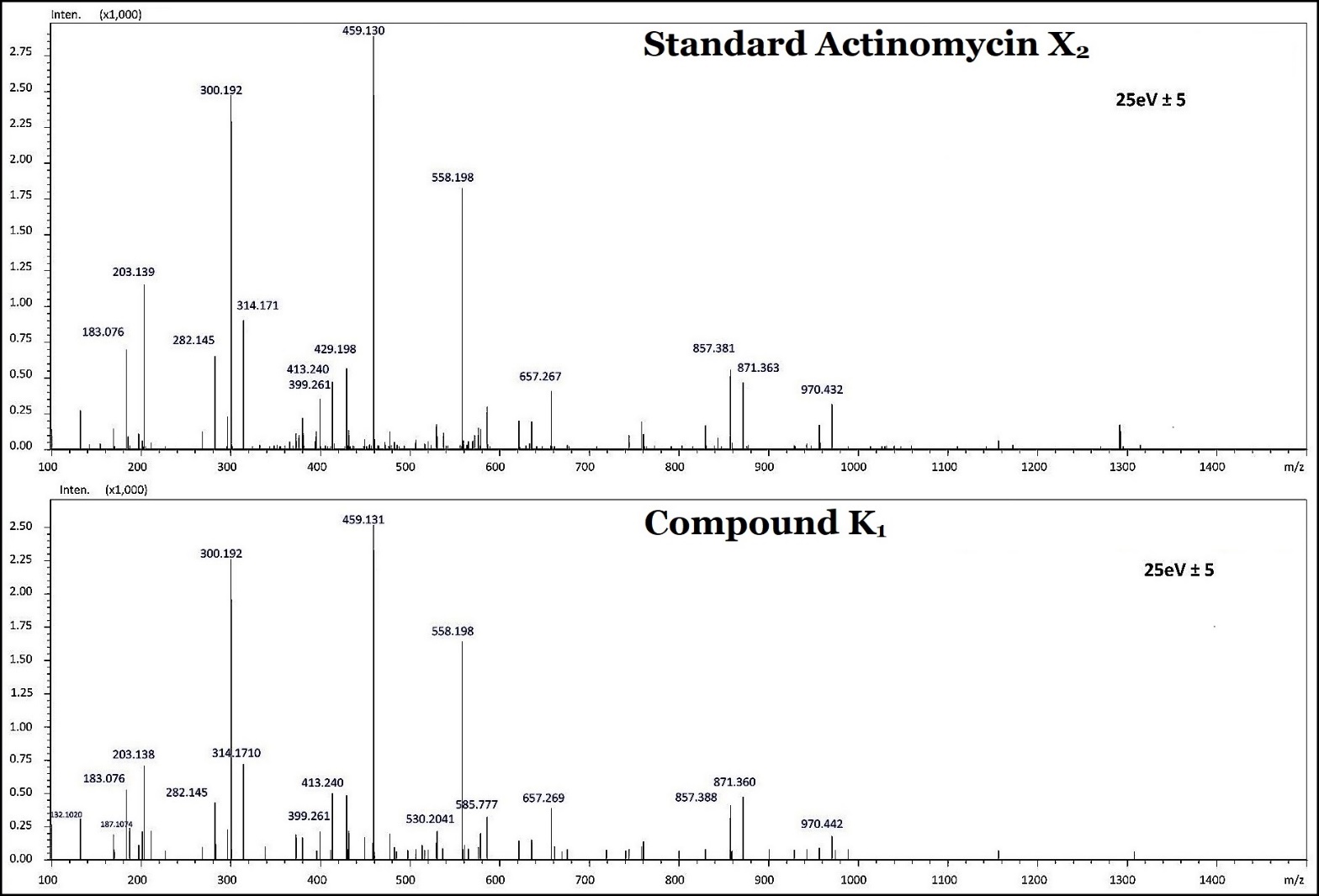


Figure S10. Comparison of LC-MS-MS (MS^2^) spectra of compound K_1_ with standard Actinomycin X_2_.


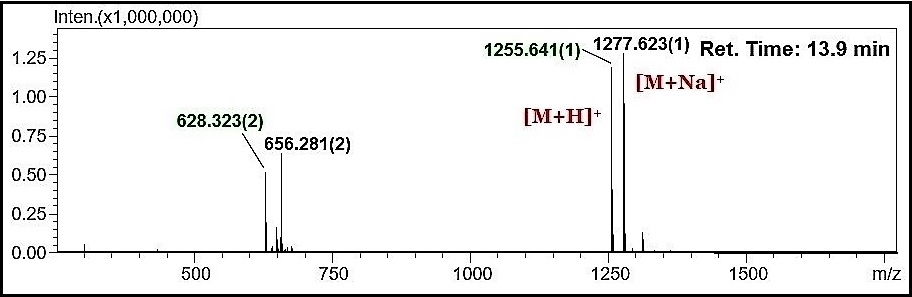


**Figure S11.** HR-ESIMS Spectra of compound K_2_.


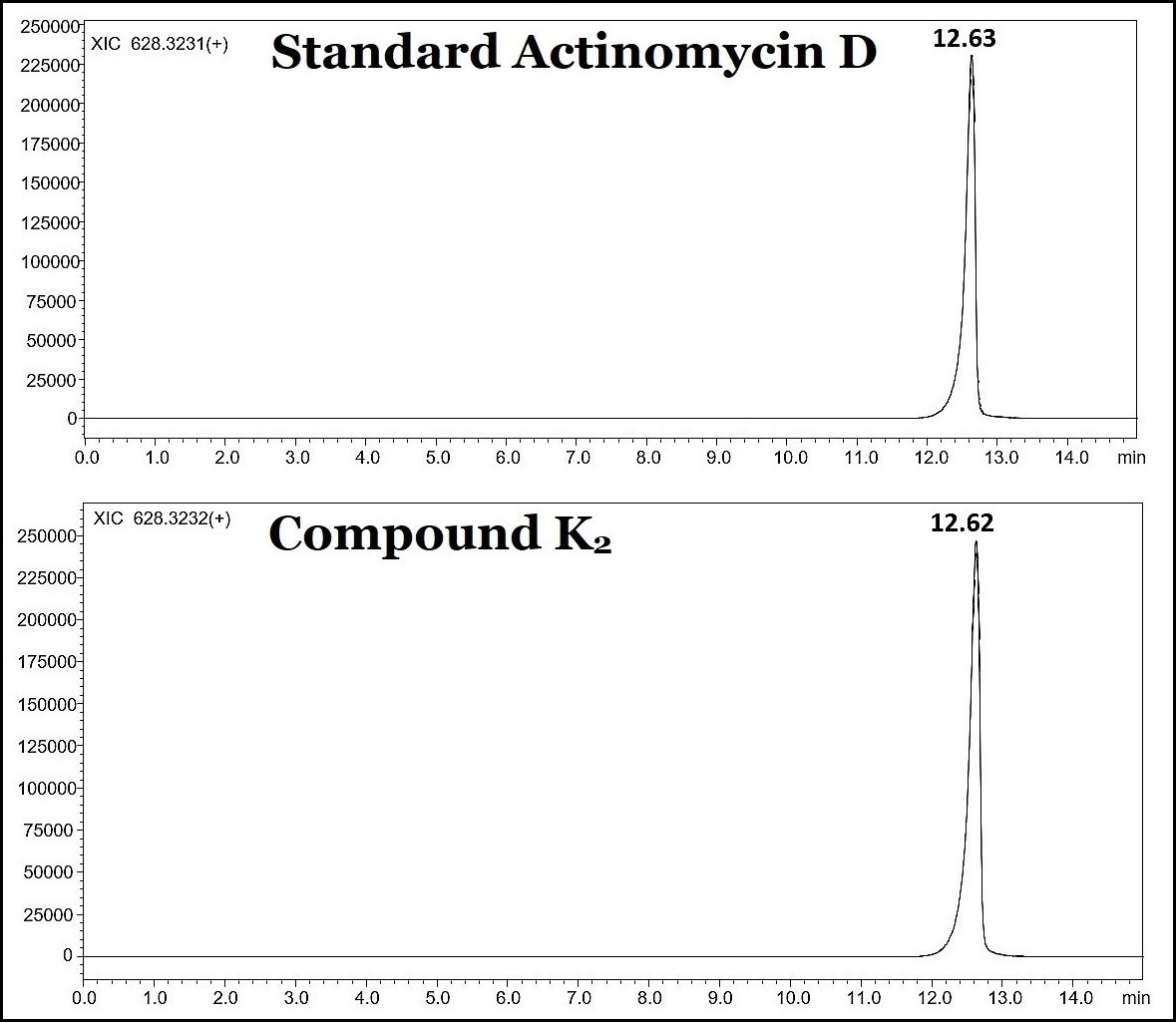


**Figure S12.** LC-MS comparison (retention time) of compound K_2_ with standard Actinomycin D.


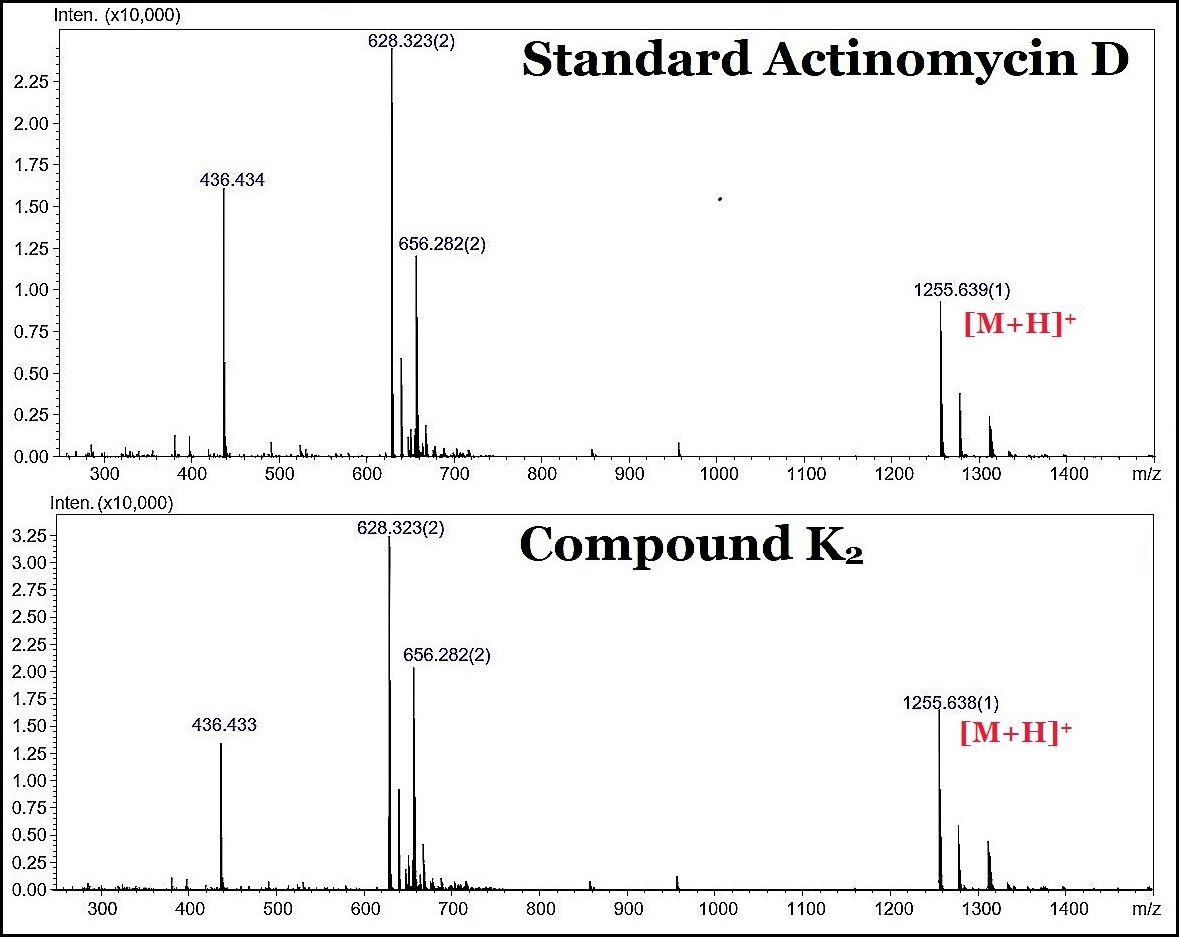


**Figure S13.** Comparison of HR-ESIMS spectra of compound K_2_ with standard Actinomycin D.


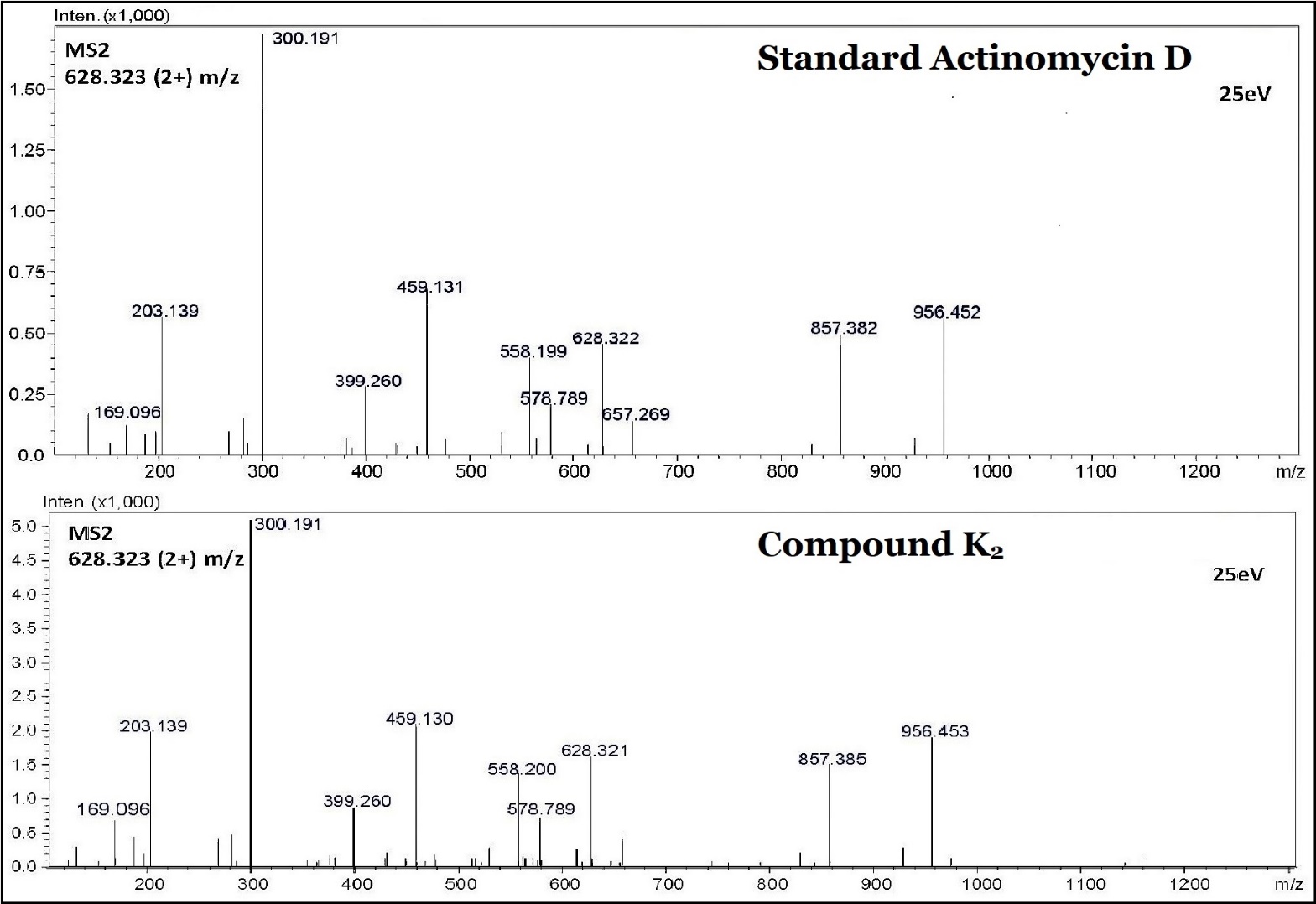


**Figure S14.** Comparison of LC-MS-MS (MS^2^) spectra of compound K_2_ with standard Actinomycin D.


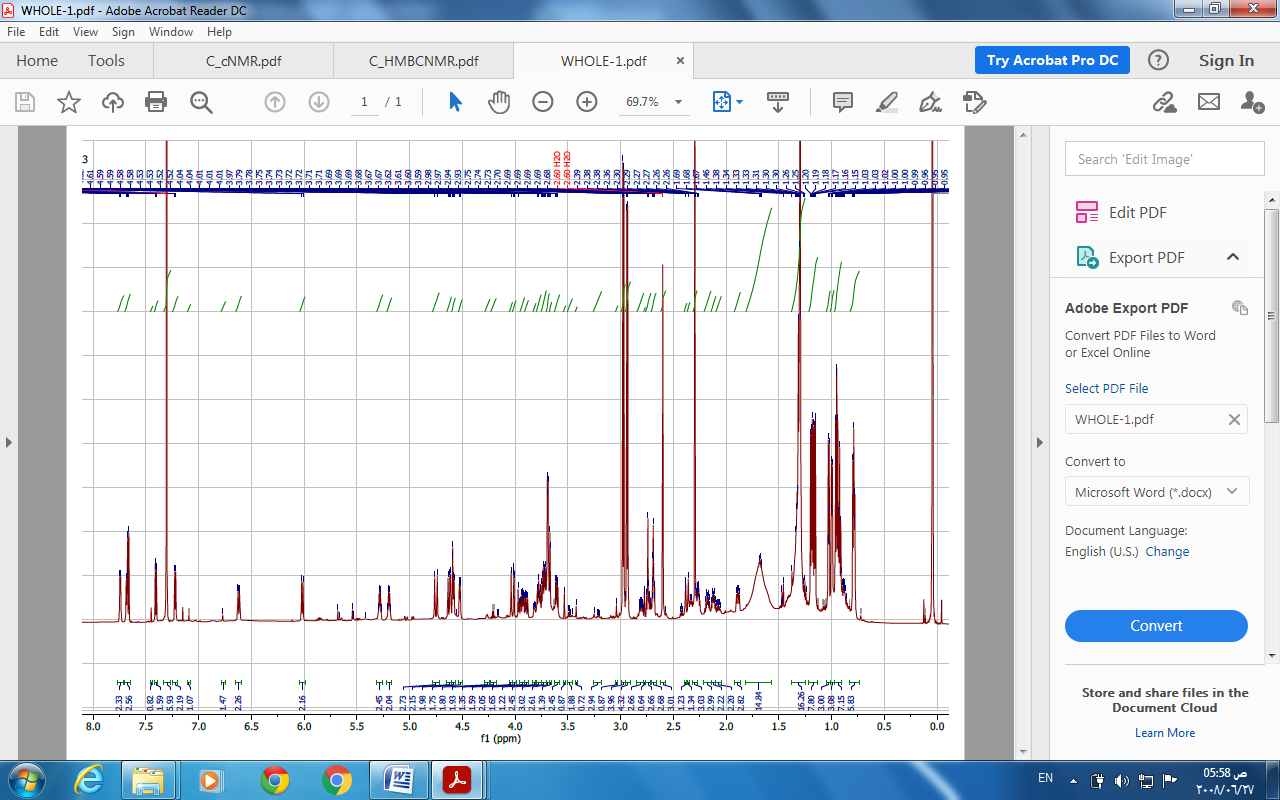


**(a)**


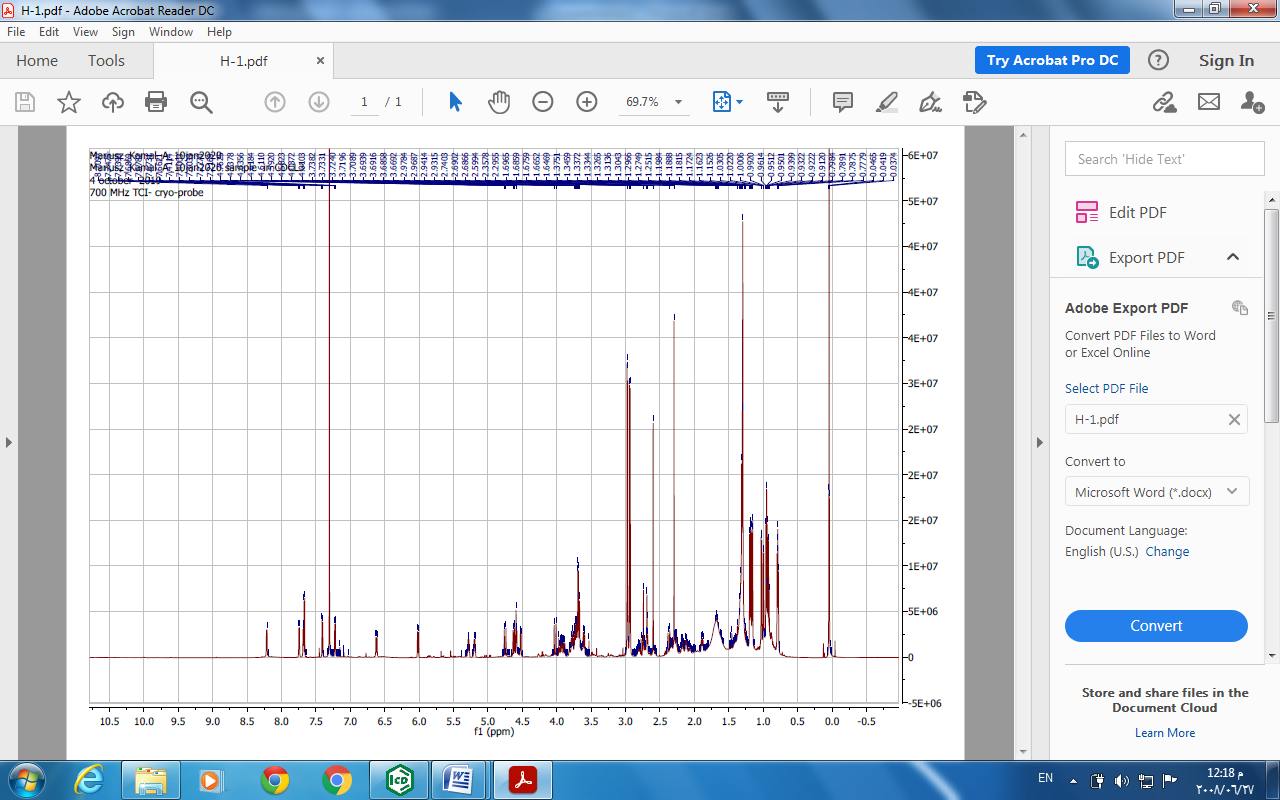


**(b)**

**Figure S15.** ^1^H NMR spectrum of compound K_1_ (*CDCl*_3_, 700 MHz).


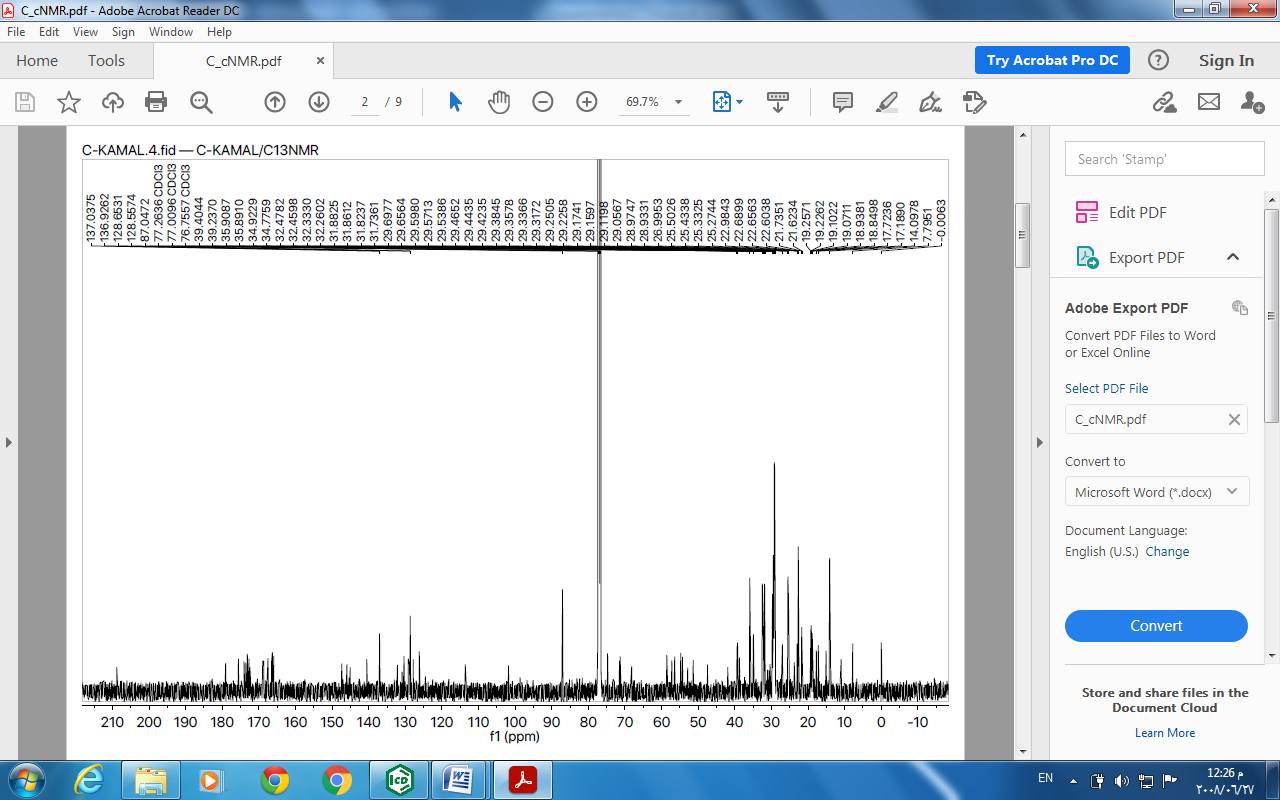


**(a)**


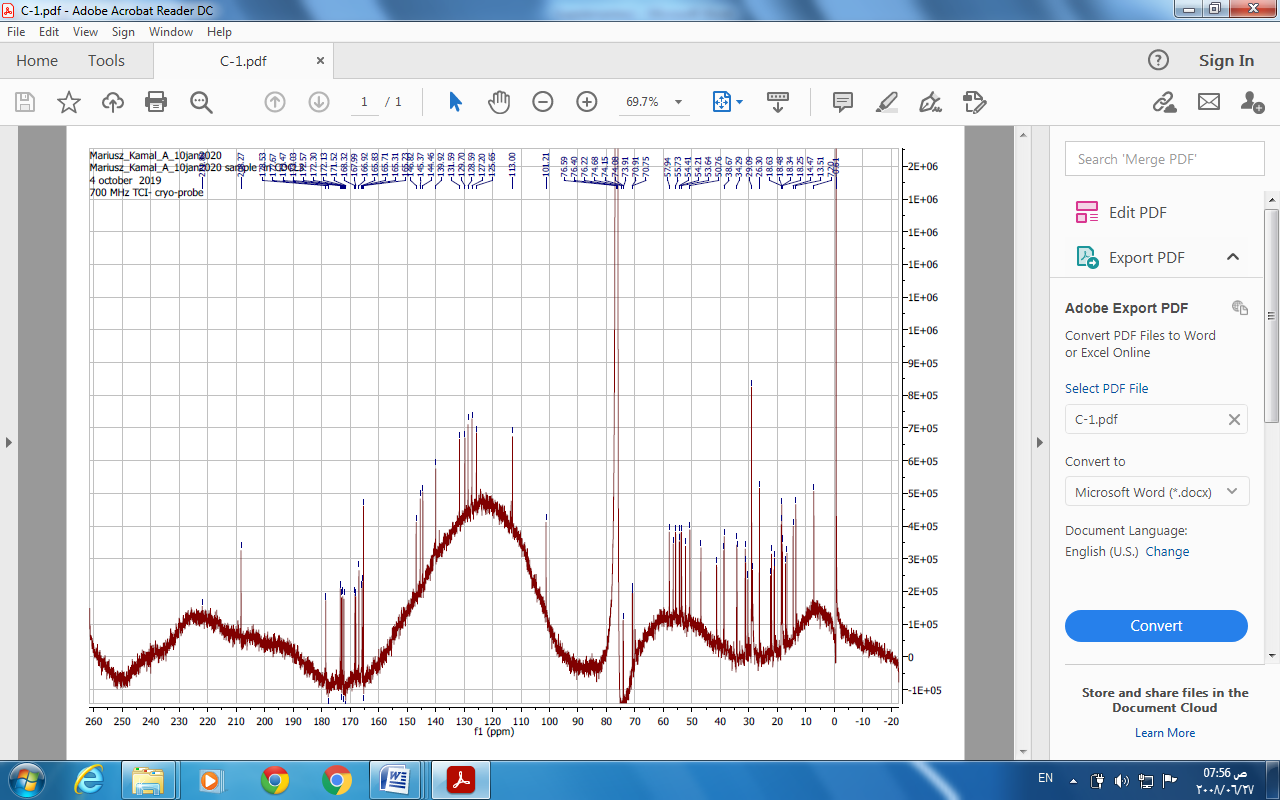


**(b)**

**Figure S16.** ^13^C NMR spectrum of compound K_1_ (*CDCl*_3_, 700 MHz).


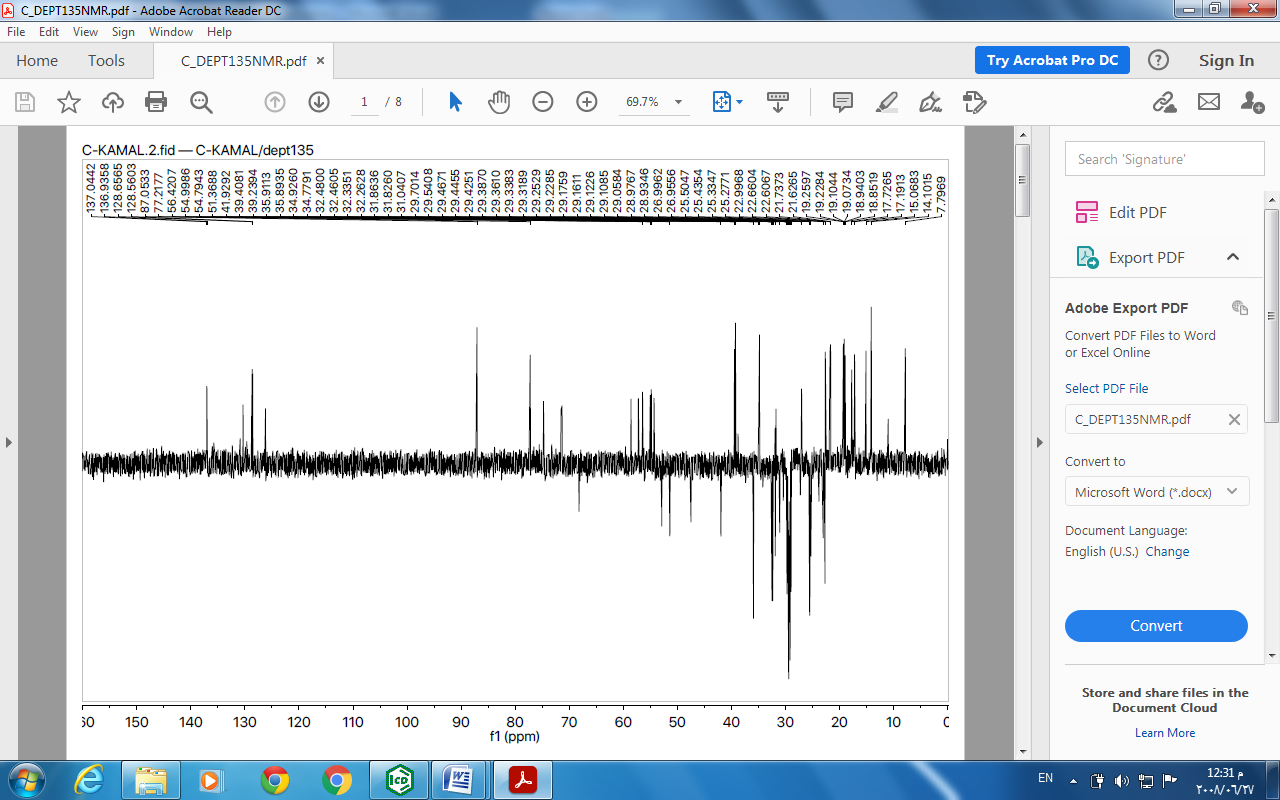


**Figure S17.** ^13^C-DEPT-135 NMR spectrum of compound K_1_ (*CDCl*_3_, 700 MHz).


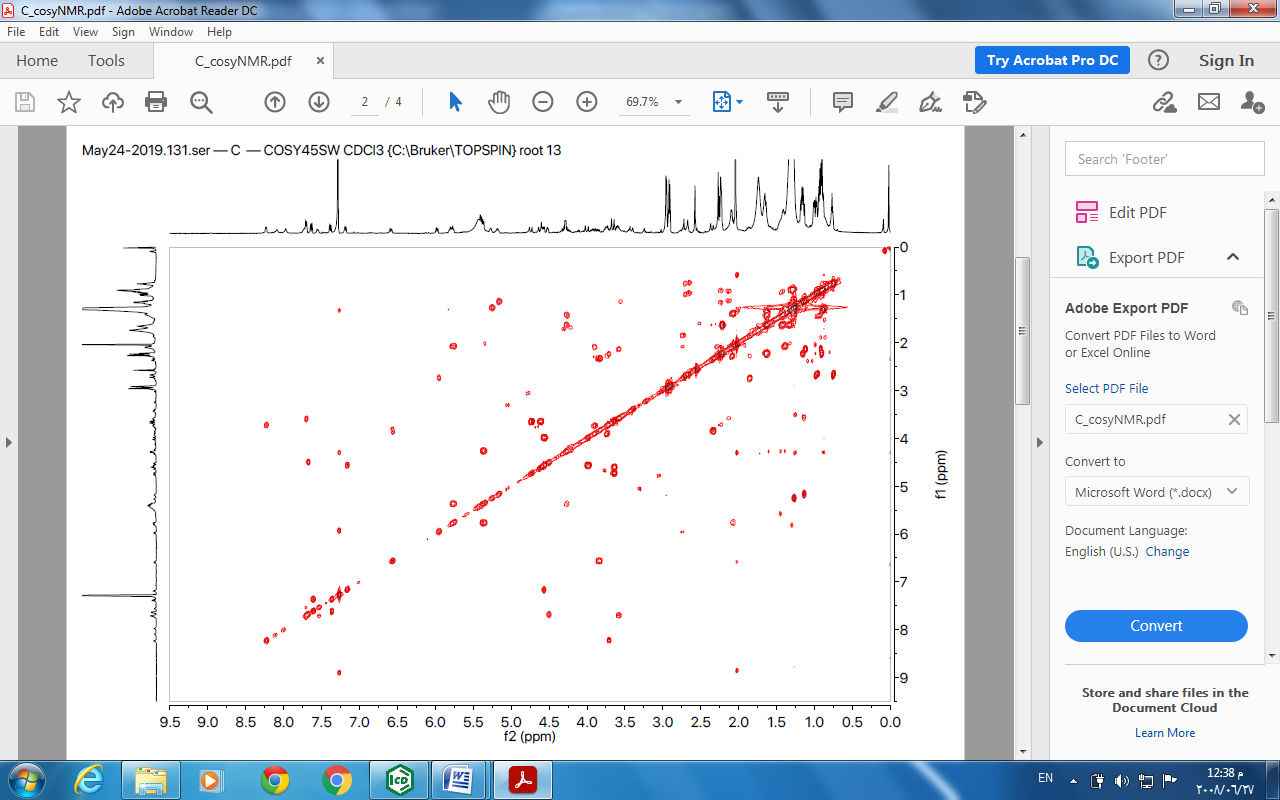


**Figure S18.** COSY spectrum of compound K_1_.


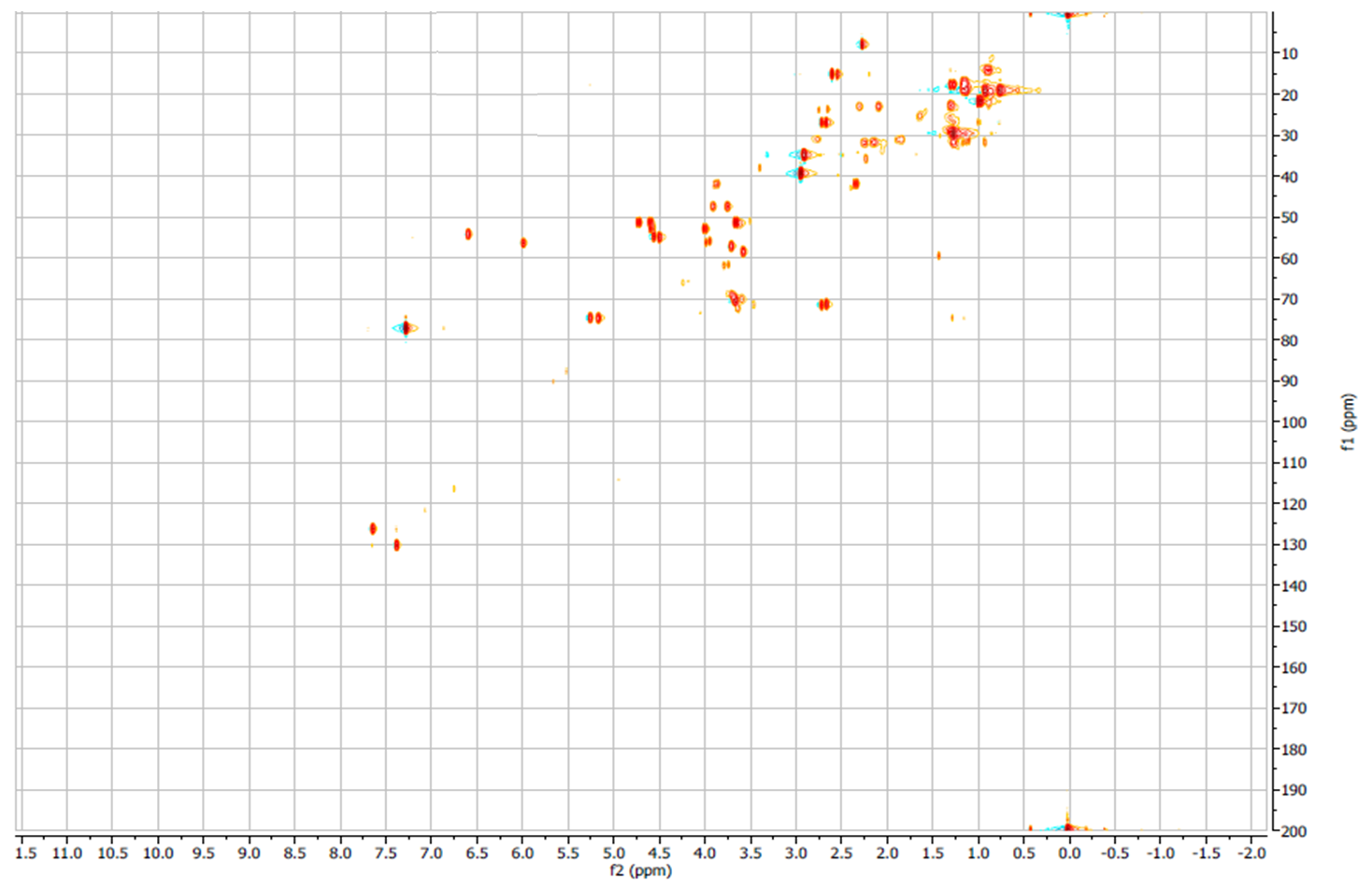


**Figure S19.** HSQC spectrum of compound K_1_.


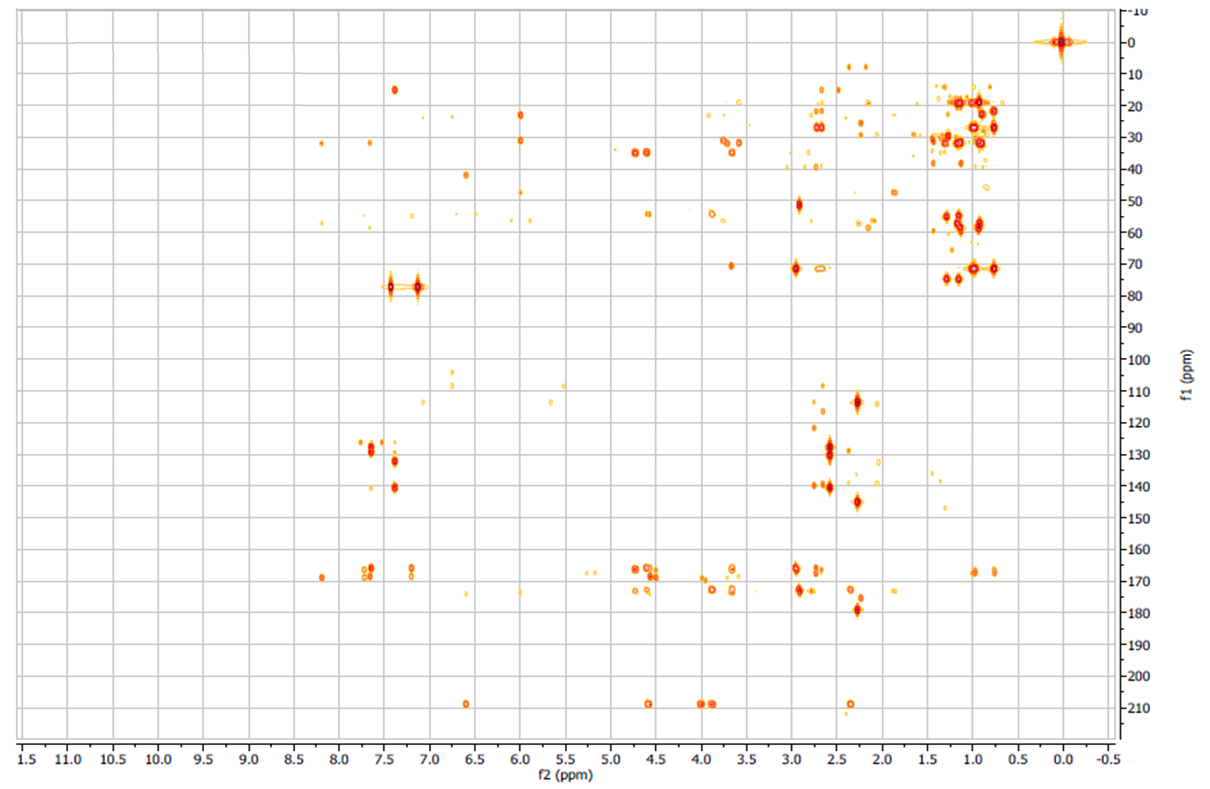


**Figure S20.** HMBC spectrum of compound K_1_.

**
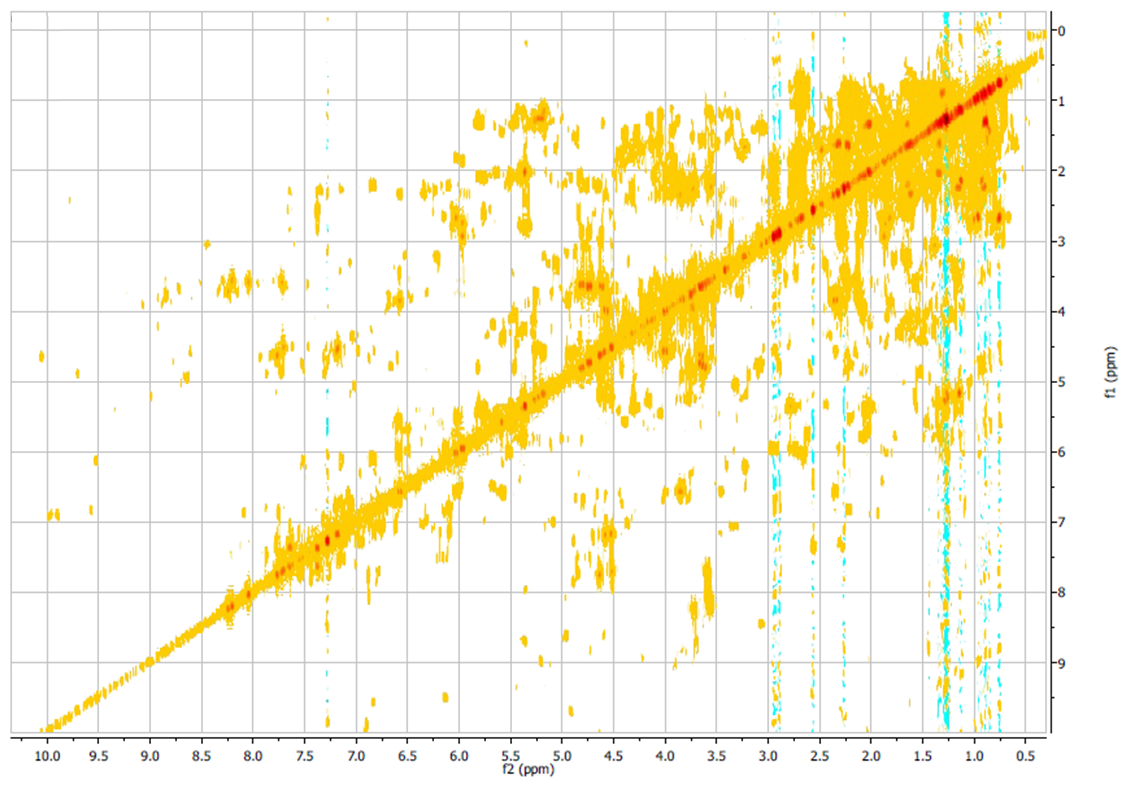
**

**Figure S21.** NOESY spectrum of compound K_1_.


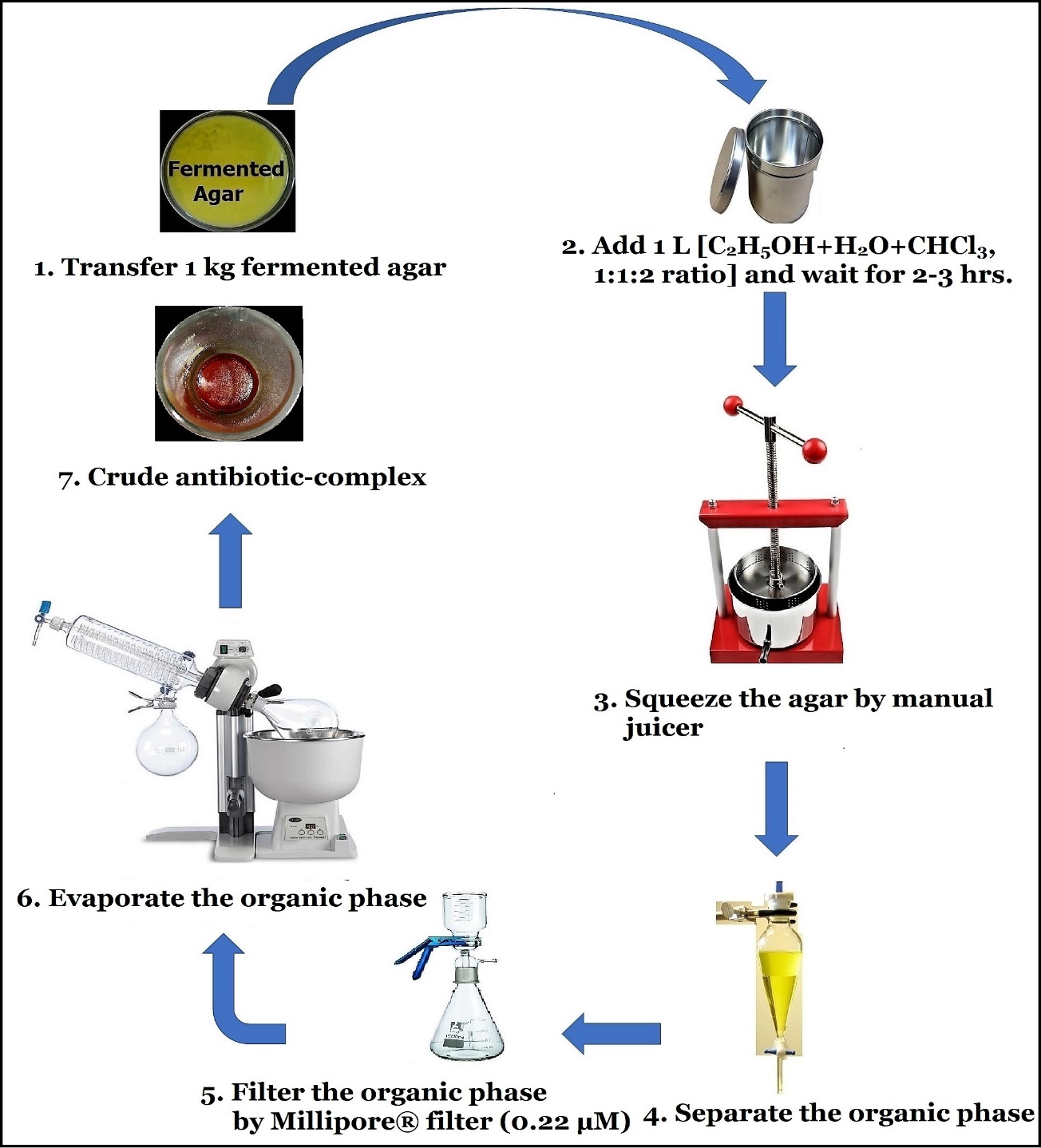


**Figure S22.** Solid-liquid extraction protocol for antibiotic extraction.

**Table S1.** Cultural characteristics of *Streptomyces smyrnaeus* UKAQ_23 on different growth media.

| **Media** | **Growth** | **Substrate mycelium color** | **Aerial mycelium color** | **Diffusible pigment** |
| --- | --- | --- | --- | --- |
| GM-1 | +++ | Canary-yellow | Yellow | No |
| GM-2 | +++ | Canary-yellow | Yellow | No |
| GM-3 | +++ | Canary-yellow | Yellow | No |
| GM-4 | +++ | Canary-yellow | Yellow | No |
| GM-5 | ++++ | Yellow | Yellow | Yellow |
| GM-6 | ++++ | Yellow | Grey | Yellow |
| GM-7 | ++++ | Yellow | Grey | Yellow |

**Note:** GM-1 = Starch soluble tryptic soy agar, GM-2 = Starch maize tryptic soy agar, GM-3 = Sucrose tryptic soy agar,

GM-4 = Glycerol tryptic soy agar, GM-5 = ISP-4 agar, GM-6 = Modified ISP-4 agar (carbon source: starch maize

and starch soluble), GM-7 = Modified ISP-4 agar (carbon source: starch maize).

“++++” indicates heavy growth, “+++” indicates moderate growth.

| **Test items** | **Results** |
| --- | --- |
| **Carbon source (1% w/v)** | |
| D (-) Mannitol | +++ |
| D (+) Dextrose | ++ |
| D (+) Galactose | + |
| Dextrin | - |
| Glycerol | ++ |
| Lactose | ++ |
| Maltose | ++ |
| Sorbitol | ++ |
| Starch maize | ++++ |
| Starch soluble | ++++ |
| Sucrose | ++++ |
| **Nitrogen source (0.5% w/v)** | |
| Ammonium chloride | + |
| Ammonium oxalate | + |
| Ammonium sulfate | ++++ |
| Cornmeal | + |
| Glycine | ++++ |
| L-Asparagine Monohydrate | ++++ |
| L-Glutamine | + |
| L-Leucine | + |
| L-Methionine | + |
| L-Tryptophan | ++ |
| Potassium nitrate | + |
| Soybean meal | +++ |
| pH tolerance  pH for optimum growth | 6-9  6.5 |
| Temperature tolerance  Optimum temperature for growth | 25-40 °C  30 °C |
| NaCl tolerance  Optimum NaCl for growth | 0.5-6.0% w/v  0.5% w/v |

**Table S2.** Physiological characteristics of *Streptomyces smyrnaeus* UKAQ_23.

**Note:** “++++” indicates excellent growth, “+++” indicates good growth, “++” indicates fair growth, “+” indicates poor

growth, “-” indicates no growth

| **Carbon source** | | **Result** | **Nitrogen source** | |
| --- | --- | --- | --- | --- |
| **Starch soluble**  **(% w/v)** | **Starch maize**  **(% w/v)** |  | **(NH_4_)_2_SO_4_ (% w/v)** | |
| 1 | 0.0 | 3.2±0.3 | 0.25 | 10.4±0.5 |
| 1 | 0.2 | 5.2±0.3 | 0.5 | 19.6±0.6 **H** |
| 1 | 0.4 | 4.8±0.1 | 0.75 | 8.3±0.7 |
| 1 | 0.6 | 4.4±0.3 | 1 | 6.8±0.6 |
| 1 | 0.8 | 4.4±0.2 | **NaCl (% w/v)** | |
| 0.0 | 1.0 | 4.0±0.2 | 0.5 | 6.4±0.4 |
| 0.2 | 1.0 | 8.0±0.6 | 0.75 | 8.4±0.3 **H** |
| 0.4 | 1.0 | 6.0±0.6 | 1 | 7.6±0.3 |
| 0.6 | 1.0 | 19.6±0.7 **H** | 1.25 | 5.5±0.1 |
| 0.8 | 1.0 | 9.6±0.4 | 1.5 | 5.0±0.2 |
| 1 | 1.0 | 6.4±0.5 | 1.75 | 3.5±0.2 |
| **K_2_HPO_4_ (% w/v)** | | | 2 | 3.0±0.2 |
| 0.3 | | 18.8±0.5 | **MgSO_4_ (% w/v)** | |
| 0.6 | | 22.4±0.4 **H** | 0.05 | 13.6±0.4 **H** |
| 0.9 | | 15.6±0.4 | 0.10 | 11.6±0.4 |
| 1.2 | | 11.1±0.3 | 0.15 | 8.4±0.3 |
| 1.5 | | 0.50±0.1 | 0.20 | 6.2±0.3 |
| **CaCl_2_ (% w/v)** | | | 0.25 | 3.9±0.3 |
| 0.05 | | 12.0±0.5 | 0.30 | 2.0±0.2 |
| 0.10 | | 14.0±0.3 | 0.35 | 1.5±0.2 |
| 0.15 | | 14.8±0.2 **H** | 0.40 | 1.0±0.2 |
| 0.20 | | 13.2±0.2 | **CaCO_3_ (% w/v)** | |
| 0.25 | | 11.5±0.5 | 0.005 | 3.2±0.2 |
| 0.30 | | 8.9±0.4 | 0.010 | 12.0±0.3 |
| 0.35 | | 6.0±0.4 | 0.015 | 24.8±0.2 **H** |
| 0.40 | | 3.8±0.7 | 0.020 | 11.5±0.3 |
| **FeSO_4_ (% w/v)** | | | 0.025 | 8.9±0.4 |
| 0.005 | | 8.8±0.3 | 0.030 | 5.8±0.2 |
| 0.010 | | 12.4±0.4 **H** | **ZnSO_4_ (% w/v)** | |
| 0.015 | | 5.6±0.4 | 0.005 | 18.4±0.5 **H** |
| 0.020 | | 3.0±0.3 | 0.010 | 15.6±0.5 |
| 0.025 | | 1.9±0.4 | 0.015 | 12.8±0.3 |
| 0.030 | | 0.9±0.2 | 0.020 | 9.8±0.2 |
| **pH** | | | 0.025 | 7.5±0.3 |
| 6.0 | | 22.0±0.5 | 0.030 | 5.2±0.3 |
| 6.5 | | 74.8±2.1 **H** | **Temperature (^o^C)** | |
| 7.0 | | 40.0±1.9 | 25 | 32.0±1.8 |
| 7.5 | | 19.0±1.3 | 30 | 74.0±2.3 |
| **Inoculum (% v/w)** | | | 35 | 255.6±1.6 **H** |
| 2.5 | | 98.5 | 40 | 101.5±1.3 |
| 5 | | 256.4 **H** | 45 | 23.0±1.1 |
| 7.5 | | 158.5 | **Incubation period (hrs.)** | |
| 10 | | 131.6 | 24 | 0.0±0.0 |
| 12.5 | | 97.6 | 48 | 0.0±0.0 |
| 15 | | 68.8 | 72 | 0.0±0.0 |
| **Agar (% w/v)** | | | 96 | 50.5±1.3 |
| 0.75 | | 46.4±0.5 | 120 | 120.3±1.8 |
| 1 | | 136.0±0.4 | 144 | 230.5±1.3 |
| 1.25 | | 199.0±1.5 | 168 | 561.3±0.3 **H** |
| 1.5 | | 256.4±1.3 **H** | 192 | 196.5±4.4 |
| 1.75 | | 98.5±1.3 | 216 | 102.4±2.7 |
| 2 | | 50.1±1.4 | 240 | 64.7±1.0 |

**Table S3.** Results of optimization of media composition (ISP-4) and fermentation conditions for maximum

antibiotic production.

**Note:** The crude antimicrobial extract yield is shown in mg/kg (Mean ± SD) of fermented agar, where "H" indicating the highest crude antimicrobial extract yield.

| **Run** | **Variables** | | | | **Yield of crude antimicrobial extract**  **(mg/kg fermented agar)** | |
| --- | --- | --- | --- | --- | --- | --- |
|  | **pH** | **Temperature (^o^C)** | **Inoculum concentration (% v/w)** | **Agar concentration (% w/v)** | **Observed values** | **Predicted values** |
| 1 | 6.5 | 30 | 7.5 | 1.5 | 376.0±0.5 | 564.0±0.0 |
| 2 | 6 | 35 | 5 | 1.75 | 421.3±0.3 | 632.0±0.0 |
| 3 | 7 | 30 | 5 | 1.5 | 421.3±1.2 | 626.0±0.0 |
| 4 | 6 | 35 | 2.5 | 1.5 | 468.7±0.4 | 703.0±0.0 |
| 5 | 6.5 | 35 | 7.5 | 1.25 | 439.3±0.3 | 659.0±0.0 |
| 6 | 6.5 | 30 | 2.5 | 1.5 | 383.3±0.4 | 575.0±0.0 |
| 7 | 6.5 | 40 | 2.5 | 1.5 | 402.0±0.5 | 603.0±0.0 |
| 8 | 6.5 | 35 | 5 | 1.5 | 561.3±0.3 | 842.0±0.0 |
| 9 | 7 | 35 | 7.5 | 1.5 | 473.3±0.3 | 710.0±0.0 |
| 10 | 6.5 | 35 | 2.5 | 1.25 | 446.7±0.6 | 670.0±0.0 |
| 11 | 6 | 35 | 7.5 | 1.5 | 461.3±0.3 | 692.0±0.0 |
| 12 | 6.5 | 35 | 5 | 1.5 | 561.3±0.3 | 842.0±0.0 |
| 13 | 6.5 | 35 | 5 | 1.5 | 561.3±1.2 | 842.0±0.0 |
| 14 | 6.5 | 35 | 2.5 | 1.75 | 399.3±0.5 | 599.0±0.0 |
| 15 | 6.5 | 30 | 5 | 1.75 | 336.0±0.4 | 504.0±0.0 |
| 16 | 7 | 35 | 2.5 | 1.5 | 480.7±0.6 | 721.0±0.0 |
| 17 | 6.5 | 30 | 5 | 1.25 | 383.3±0.8 | 575.0±0.0 |
| 18 | 7 | 35 | 5 | 1.25 | 480.7±1.2 | 721.0±0.0 |
| 19 | 7 | 40 | 5 | 1.5 | 436.0±0.5 | 654.0±0.0 |
| 20 | 6 | 40 | 5 | 1.5 | 424.0±0.3 | 636.0±0.0 |
| 21 | 6.5 | 40 | 7.5 | 1.5 | 394.7±0.5 | 592.0±0.0 |
| 22 | 6.5 | 40 | 5 | 1.75 | 354.7±0.6 | 532.0±0.0 |
| 23 | 6 | 30 | 5 | 1.5 | 405.3±0.3 | 608.0±0.0 |
| 24 | 6.5 | 40 | 5 | 1.25 | 402.0±0.5 | 603.0±0.0 |
| 25 | 7 | 35 | 5 | 1.75 | 433.3±1.2 | 650.0±0.0 |
| 26 | 6 | 35 | 5 | 1.25 | 468.7±0.6 | 703.0±0.0 |
| 27 | 6.5 | 35 | 7.5 | 1.75 | 392.0±0.5 | 588.0±0.0 |

**Table S4.** BBD using 3-levels-4-variables showing observed and predicted responses.

| ***m/z* of fragment K_1_** | **Modified fragments** | **Fragments corresponding to Actinomycin D** |
| --- | --- | --- |
| 970.442 | B_2_+14 |  |
| 871.360 | B_1_+14 |  |
| 857.388 |  | B_1_ |
| 657.269 |  | V+Val |
| 558.198 |  | V |
| 459.131 |  | VI |
| 413.240 | Y_4_+14 |  |
| 399.281 |  | Y_4_ |
| 314.171 | Y_3_+14 |  |
| 300.192 |  | Y_3_ |
| 282.145 | HVal-Pro-Ser^+^ +14 |  |
| 203.145 |  | Y_2_ |
| 183.076 | HPro-Sar^+^ +14 |  |

**Table S5.** Comparison of CID MS/MS fragments of K_1_ with Actinomycin D.

**Note:** The nomenclature of fragment ions is according to reference^17^.

| ***m/z* of K_2_ fragment** | ***m/z* of std. Actinomycin D fragment**^17^ | **Assignment of fragment**^17^ |
| --- | --- | --- |
| 956.453 | 956.4 | [MH^+^ - (Pro-Sar-MeVal)] |
| 857.385 | 875.1 | [MH ^+^ - (Val-Pro-MeVal)] |
| 657.269 | 657.0 | [V + Val] |
| 628.321 | 629.4 | [V + Val - CO] |
| 558.200 | 558.1 | V |
| 459.130 | 459.0 | VI |
| 399.260 | 399.1 | Y4 |
| 300.191 | 300.1 | Y3 |
| 203.139 | 202.9 | Y2 |
| 169.096 | 168.9 | [H-Pro-Sar]^+^ |

**Table S6.** Comparison of CID MS/MS fragmentation spectrum of compound K_2_ with Actinomycin D*

**Note:** The nomenclature of fragment ions is according to reference^17^.

| **Position** | **^1^H (*J* in Hz)** | **^13^C** | **DEPT** | **COSY** | **HMBC** |
| --- | --- | --- | --- | --- | --- |
| **α-Ring** | | | | | |
| **Thr** |  |  |  |  |  |
| **1** | - | 168.32 | C | - | - |
| **2** | 4.52, dd, 6.0, 2.7 | 54.42 | CH | NH | C-14, Thr C-1 |
| **3** | 5.29, qd, 6.3, 2.7 | 74.08 | CH | H-4 | MeVal C-1 |
| **4** | 1.30, d, 6.5 | 17.17 | CH_3_ | H-3 | Thr C-2, 3 |
| **NH** | 7.22, d, 7.0 | - | - | Thr H-2 | C-14, Thr C-1 |
| **Val** |  |  |  |  |  |
| **1** | - | 173.03 | C | - | - |
| **2** | 3.72, dd, 9.4, 6.3 | 56.59 | CH | H-3 | Thr C-1, Val C-1, 3 |
| **3** | 2.26, m | 31.28 | CH | H-2, 4, 5 | Val C-1, 2 |
| **4** | 1.19, d, 6.7 | 18.26 | CH_3_ | H-3 | Val C-2, 3, 5 |
| **5** | 0.94, d, 7.1 | 18.65 | CH_3_ | H-3 | Val C-2, 3, 4 |
| **NH** | 8.21, d, 6.0 | - | - | Val H-2 | Thr C-1, Val C-2, 3 |
| **Pro** |  |  |  |  |  |
| **1** | - | 172.57 | C | - | - |
| **2** | 6.02, d, 9.2 | 55.73 | CH | H-3b | Val C-1, 3, 4, 5 |
| **3** | 1.88, dd, 11.6, 6.8  2.80, m | 30.42 | CH_2_ | H-3b  H-2, 3a | Pro C-4, 5  Pro C-1, 2, 4 |
| **4** | 2.12, m  2.32, m | 22.41 | CH_2_ | H-4b, 5b  H-4a, 5a | Pro C-2 |
| **5** | 3.78, d, 9.4  3.94, dd, 11.2, 4.3 | 46.85 | CH_2_ | H-4b, 5b  H-4a, 5a | Pro C-3 |
| **Sar** |  |  |  |  |  |
| **1** | - | 165.31 | C | - | - |
| **2** | 4.75, d, 17.2  3.69, d, 17.2 | 50.76 | CH_2_ | H-2b  H-2a | Sar C-1, NMe, Pro C-1  Sar C-1, NMe, Pro C-1 |
| **NMe** | 2.88, s | 34.30 | CH_3_ |  | Sar C-1, 2, Pro C-1 |
| **MeVal** |  |  |  |  |  |
| **1** | - | 165.83 | C | - | - |
| **2** | 2.68, m | 70.75 | CH |  | Sar C-1, MeVal C-3, 4, 5, NMe |
| **3** | 2.68, m | 26.31 | CH | H-4, 5 | MeVal C-1, 2 |
| **4** | 1.00, d, 6.0 | 21.04 | CH_3_ | H-3 | MeVal C-2, 3, 5 |
| **5** | 0.79, d, 6.5 | 18.35 | CH_3_ | H-3 | MeVal C-2, 3, 4 |
| **NMe** | 2.94, s | 38.68 | CH_3_ |  | Sar C-1, MeVal C-2 |
| **β-Ring** | | | | | |
| **Thr** |  |  |  |  |  |
| **1** | - | 167.99 | C | - | - |
| **2** | 4.58, dd, 6.0, 2.7 | 54.21 | CH | NH | C-11, Thr C-1 |
| **3** | 5.20, qd, 6.2, 2.7 | 74.16 | CH | H-4 | MeVal C-1 |
| **4** | 1.17, d, 6.3 | 16.65 | CH_3_ | H-3 | Thr C-2, 3 |
| **NH** | 7.74, d, 5.9 | - | - | Thr H-2 | C-11, Thr C-1 |
| **Val** |  |  |  |  |  |
| **1** | - | 173.47 | C | - | - |
| **2** | 3.61, dd, 9.5, 6.0 | 57.95 | CH | H-3 | Thr C-1, Val C-3, 5 |
| **3** | 2.18, m | 31.15 | CH | H-2, 4, 5 | Val C-2 |
| **4** | 1.15, d, 6.7 | 18.40 | CH_3_ | H-3 | Val C-2, 3, 5 |
| **5** | 0.95, d, 7.1 | 18.63 | CH_3_ | H-3 | Val C-2, 3, 4 |
| **NH** | 7.68, d, 6.0 | - | - | Val H-2 | Thr C-1, Val C-2, 3 |
| **OxoPro** |  |  |  |  |  |
| **1** | - | 172.13 | C | - | - |
| **2** | 6.62, dd, 10.5, 1.1 | 53.64 | CH | H-3a | Val C-1, OxoPro C-3, 4 |
| **3** | 3.90, dd, 17.2, 10.6  2.36, d, 17.8 | 41.34 | CH_2_ | H-2, 3b  H-3a | OxoPro C-1, 2, 4  OxoPro C-1, 4 |
| **4** | - | 208.27 | C | - | - |
| **5** | 4.61, d, 18.4  3.97, m | 52.27 | CH_2_ | H-5b  H-5a | OxoPro C-2, 4  OxoPro C-4 |
| **Sar** |  |  |  |  |  |
| **1** | - | 165.71 | C | - | - |
| **2** | 4.64, d, 17.2  3.68, d, 17.2 | 50.72 | CH_2_ | H-2b  H-2a | Sar C-1, NMe, Pro C-1  Sar C-1, NMe, Pro C-1 |
| **NMe** | 2.89, s | 34.16 | CH_3_ |  | Sar C-1, 2, Pro C-1 |
| **MeVal** |  |  |  |  |  |
| **1** | - | 166.88 | C | - | - |
| **2** | 2.73, m | 70.91 | CH |  | Sar C-1, MeVal C-3, 4, NMe |
| **3** | 2.73, m | 26.31 | CH | H-4, 5 | MeVal C-1, 2, NMe |
| **4** | 1.03, d, 5.9 | 21.15 | CH_3_ | H-3 | MeVal C-2, 3, 5 |
| **5** | 0.78, d, 7.6 | 18.50 | CH_3_ | H-3 | MeVal C-2, 3, 4 |
| **NMe** | 2.93, s | 38.84 | CH_3_ |  | Sar C-1, MeVal C-2 |
| **Chromophore** | | | | | |
| **1** | - | 101.21 | C | - | - |
| **2** | - | 146.82 | C | - | - |
| **3** | - | 178.53 | C | - | - |
| **4** | - | 113.00 | C | - | - |
| **4a** | - | 144.46 | C | - | - |
| **5a** | - | 139.93 | C | - | - |
| **6** | - | 127.20 | C | - | - |
| **7** | 7.40, d, 7.7 | 129.70 | CH | H-8 | C-5a, 9, 13 |
| **8** | 7.66, d, 7.7 | 125.65 | CH | H-7 | C-6, 9a, 14 |
| **9** | - | 131.59 | C | - | - |
| **9a** | - | 128.60 | C | - | - |
| **10a** | - | 145.37 | C | - | - |
| **11** | - | 166.92 | C | - | - |
| **12** | 2.29, s | 7.20 | CH_3_ |  | C-3, 4, 4a |
| **13** | 2.59, s | 14.48 | CH_3_ |  | C-5a, 6, 7 |
| **14** | - | 165.31 | C | - | - |

**Table S7.** 1D and 2D NMR data of compound K_1_ (*CDCl*_3_, 700 MHz)

| **Test Organisms** | **Mean ± SD Zone of inhibition (mm)** | | | **Levofloxacin (5µg/disc)** |
| --- | --- | --- | --- | --- |
|  | **X_2_ (5µg/disc)** | **D (5µg/disc)** | **X_2_ + D (1:1, ratio) (5µg/disc)** |  |
| **Non-MRSA bacteria** | | | | |
| *S.aureus* ATCC 29213 | 14.8 ± 0.20 | 8.2 ± 0.10 | 13.0 ± 0.10 | 35.6 ± 0.20 |
| *S.saptophyticus* ATCC 43867 | 12.3 ± 0.30 | 6.5 ± 0.10 | 11.7 ± 0.17 | 26.2 ± 0.17 |
| *S.epidermidis* ATCC 12228 | 8.5 ± 0.17 | 6.6 ± 0.17 | 7.3 ± 0.17 | 21.6 ± 0.26 |
| *S.pyogenes*-A ATCC 27736 | 16.5 ± 0.17 | 12.2 ± 0.10 | 15.6 ± 0.17 | 20.3 ± 0.17 |
| *S.pneumoniae* ATCC 49619 | 15.8 ± 0.26 | 11.9 ± 0.10 | 14.6 ± 0.10 | 18.7 ± 0.17 |
| *E.faecalis* ATCC 29212 | 16.7 ± 0.17 | 12.4 ± 0.10 | 16.7 ± 0.10 | 21.9 ± 0.10 |
| *B.cereus* ATCC 10876 | 17.9 ± 0.17 | 13.2 ± 0.17 | 17.3 ± 0.10 | 34.6 ± 0.10 |
| **MRSA bacteria** | | | | |
| MRSA-A* | 14.8 ± 0.26 | 9.3 ± 0.20 | 11.6 ± 0.10 | 29.0 ± 0.20 |
| MRSA-B* | 13.9 ± 0.17 | 12.4 ± 0.10 | 15.4 ± 0.10 | 19.5 ± 0.17 |
| MRSA-C* | 15.5 ± 0.26 | 6.5 ± 0.10 | 11.6 ± 0.10 | 33.9 ± 0.10 |

**Table S8.** Primary antimicrobial activity of isolated actinomycins (X_2_, D and X_2_ + D)

*Clinical isolates

| **Fermentation Media** | **Carbon source (1% w/v)** | **Nitrogen source (0.5% w/v)** |
| --- | --- | --- |
| FM-1 | Sucrose | Ammonium sulphate |
| FM-2 | Sucrose | Glycine |
| FM-3 | Sucrose | L-Asparagine Monohydrate |
| FM-4 | Sucrose | Soya meal |
| FM-5 | Starch soluble | Ammonium sulphate |
| FM-6 | Starch soluble | Glycine |
| FM-7 | Starch soluble | L-Asparagine Monohydrate |
| FM-8 | Starch soluble | Soya meal |
| FM-9 | Starch maize | Ammonium sulphate |
| FM-10 | Starch maize | Glycine |
| FM-11 | Starch maize | L-Asparagine Monohydrate |
| FM-12 | Starch maize | Soya meal |
| FM-13 | Glycerol | Ammonium sulphate |
| FM-14 | Glycerol | Glycine |
| FM-15 | Glycerol | L-Asparagine Monohydrate |
| FM-16 | Glycerol | Soya meal |
| FM-17 | D (-) Mannitol | Ammonium sulphate |
| FM-18 | D (-) Mannitol | Glycine |
| FM-19 | D (-) Mannitol | L-Asparagine Monohydrate |
| FM-20 | D (-) Mannitol | Soya meal |
| FM-21 | D (+) Dextrose | Ammonium sulphate |
| FM-22 | D (+) Dextrose | Glycine |
| FM-23 | D (+) Dextrose | L-Asparagine monohydrate |
| FM-24 | D (+) Dextrose | Soya meal |

**Table S9.** Various fermentation media used in antibiotic production.

| **Levels** | **Variables** | | | |
| --- | --- | --- | --- | --- |
|  | **pH** | **Temperature (^o^C)** | **Inoculum concentration**  **(% v/w)** | **Agar concentration**  **(% w/v)** |
| -1 | 6 | 30 | 2.5 | 1.25 |
| 0 | 6.5 | 35 | 5 | 1.5 |
| 1 | 7 | 40 | 7.5 | 1.75 |

**Table S10.** Coded and actual values of the variables tested in the BBD

| **Run** | **Variables** | | | |
| --- | --- | --- | --- | --- |
|  | **pH** | **Temperature (^o^C)** | **Inoculum concentration**  **(% v/w)** | **Agar concentration**  **(% w/v)** |
| 1 | 0 | -1 | 1 | 0 |
| 2 | -1 | 0 | 0 | 1 |
| 3 | 1 | -1 | 0 | 0 |
| 4 | -1 | 0 | -1 | 0 |
| 5 | 0 | 0 | 1 | -1 |
| 6 | 0 | -1 | -1 | 0 |
| 7 | 0 | 1 | -1 | 0 |
| 8 | 0 | 0 | 0 | 0 |
| 9 | 1 | 0 | 1 | 0 |
| 10 | 0 | 0 | -1 | -1 |
| 11 | -1 | 0 | 1 | 0 |
| 12 | 0 | 0 | 0 | 0 |
| 13 | 0 | 0 | 0 | 0 |
| 14 | 0 | 0 | -1 | 1 |
| 15 | 0 | -1 | 0 | 1 |
| 16 | 1 | 0 | -1 | 0 |
| 17 | 0 | -1 | 0 | -1 |
| 18 | 1 | 0 | 0 | -1 |
| 19 | 1 | 1 | 0 | 0 |
| 20 | -1 | 1 | 0 | 0 |
| 21 | 0 | 1 | 1 | 0 |
| 22 | 0 | 1 | 0 | 1 |
| 23 | -1 | -1 | 0 | 0 |
| 24 | 0 | 1 | 0 | -1 |
| 25 | 1 | 0 | 0 | 1 |
| 26 | -1 | 0 | 0 | -1 |
| 27 | 0 | 0 | 1 | 1 |

**Table S11.** Box-Behnken design (BBD) for the optimization of antibiotic production by *Streptomyces smyrnaeus* UKAQ_23
